# Identification of QTLs for wheat heading time across multiple-environments

**DOI:** 10.1007/s00122-022-04152-6

**Published:** 2022-07-01

**Authors:** Salma Benaouda, Said Dadshani, Patrice Koua, Jens Léon, Agim Ballvora

**Affiliations:** 1 Institute for Crop Science and Resource Conservation, Chair of Plant Breeding, Rheinische Friedrich-Wilhelms-University, Katzenburgweg 5, 53115 Bonn, Germany; 2 Field Lab Campus Klein-Altendorf, Rheinische Friedrich-Wilhelms-University, Bonn, Germany

## Abstract

**Key message:**

The genetic response to changing climatic factors selects consistent across the tested environments and location-specific thermo-sensitive and photoperiod susceptible alleles in lower and higher altitudes, respectively, for starting flowering in winter wheat.

**Abstract:**

Wheat breeders select heading date to match the most favorable conditions for their target environments and this is favored by the extensive genetic variation for this trait that has the potential to be further explored. In this study, we used a germplasm with broad geographic distribution and tested it in multi-location field trials across Germany over three years. The genotypic response to the variation in the climatic parameters depending on location and year uncovered the effect of photoperiod and spring temperatures in accelerating heading date in higher and lower latitudes, respectively. Spring temperature dominates other factors in inducing heading, whereas the higher amount of solar radiation delays it. A genome-wide scan of marker-trait associations with heading date detected two QTL: an adapted allele at locus TaHd102 on chromosome 5A that has a consistent effect on HD in German cultivars in multiple environments and a non-adapted allele at locus TaHd044 on chromosome 3A that accelerates flowering by 5.6 days. TaHd102 and TaHd044 explain 13.8% and 33% of the genetic variance, respectively. The interplay of the climatic variables led to the detection of environment specific association responding to temperature in lower latitudes and photoperiod in higher ones. Another locus TaHd098 on chromosome 5A showed epistatic interactions with 15 known regulators of flowering time when non-adapted cultivars from outside Germany were included in the analysis.

**Supplementary Information:**

The online version contains supplementary material available at 10.1007/s00122-022-04152-6.

## Introduction

Heading date (HD), representing the initiation of flowering time, is one of the most targeted and extensively studied traits in breeding programs designed to improve yield stability under various climatic conditions. Plants capable of adapting to extreme climates can avoid inappropriate stress factors such as frost, heat, and drought by adjusting their flowering time to seasonal conditions to protect the floral organs (Fjellheim et al. 2014). Such an adaptive mechanism of controlling the timing of starting the transition from vegetative to reproductive phase is a useful tool for selecting cultivars that match different environments and geographical regions and even to adapt regional cultivars to coming climate change scenarios (Guedira et al. 2016).

Wheat (*Triticum aestivum* L*.*) is a leading food grain crop and a staple source of nutrients for around 40% of the world’s population (FAO 2019). The adaptability of wheat to wide climatic regimes is derived from large natural variation in heading date, generated by allelic diversity in the genes regulating growth and developmental stages including flowering time (Worland 2001). Three distinct pathways interact to control flowering time in wheat: vernalization, photoperiod, and earliness per se. The group of four vernalization (*VRN)* genes regulates the molecular mechanisms for the requirement of vernalization and exposure to cold in wheat: *VRN1* and its paralog *VRN-D4* encode *MADS-box* proteins with high similarity to *Arabidopsis thaliana* meristem identity *APETALA1 (AP1),* whereas *VRN2* locus includes two tandemly duplicated genes *ZCCT1* and *ZCCT2* (Yan et al. 2006). These genes encode for proteins carrying a putative zinc finger and a *CCT* domain referred to *CONSTANS (CO), CONSTANS-like (COL),* and *TIMING OF CAB1 (TOC1)* (Putterill et al. 1995; Strayer et al. 2000; Robson et al. 2001). *VRN3* is a homolog of the *Arabidopsis* photoperiod gene *FLOWERING LOCUS T* (Yan et al. 2006). Natural allelic variation in one or more of the *VRN* genes leads to the differentiation between winter and spring growth habits. Hexaploid wheat bearing a dominant allele at *VRN1* or *VRN3* loci requires less cold treatment to flower (spring habit), while the presence of recessive alleles at these loci increases the demand for vernalization (winter habit) (Turner et al. 2013). *VRN2* genes are dominant in winter growth habit (Yan et al. 2004a, b). Winter bread wheat is a long day plant and a photoperiod sensitive crop that flowers after accumulation of a critical day length (Fjellheim et al. 2014). The day length responsive gene, *PPD-D1*, is an ortholog of pseudo-response regulator *(PRR)* of *Arabidopsis* in wheat (Beales et al., 2007; Turner et al., 2005). The semi-dominant 2,089 bp deletion upstream of the coding region in the allele *Ppd-D1a* causes insensitivity to photoperiod and accelerates flowering time (Beales et al. 2007; Shaw et al. 2012). Earliness per se* (Eps)* refers to the remaining earliness of flowering time when vernalization requirements and photoperiodic sensitivity are fulfilled (Worland, 1996). The Eps definitions suggest that these genes regulate flowering independent of environmental cues (Bullrich et al. 2002). Nevertheless, recent studies indicate that they exhibit temperature sensitivity in wheat (Ochagavía et al. 2019; Prieto et al. 2020).

Numerous strategies have been adopted to decipher the genetic control of flowering time in wheat, such as the candidate gene approach (Eagles et al. 2010; Rousset et al. 2011; Bentley et al. 2013), and the meta-QTL (quantitative trait locus) analysis, which includes individual and separate QTL studies. The last was used firstly in maize and was conducted in wheat as well using either biparental populations or collections of association panels (Hanocq et al. 2007; Griffiths et al. 2009; Kamran et al. 2014). Additionally, new technologies such as high-throughput genotyping and sequencing, and the development of powerful statistical tools based on linkage disequilibrium (LD) could be exploited in genome-wide scans (GWS). Identification of genetic and molecular interactions has improved the understanding of the mechanisms underlying complex traits (Phillips 2008). Therefore, many GWSs for marker-trait associations (MTA) have used epistatic analysis as a complementary approach to discover more genomic regions associated with intricate traits in different crops including maize, wheat, and rapeseed (Buckler et al. 2009; Liu et al. 2012a, b; Steinhoff et al. 2012; Würschum et al. 2013).

In Europe, most of the reported studies on flowering time in the field use 1st January or sowing day as the date for starting the scoring until the anthesis stage. Both dates are including the vernalization period, where the plant is facing low temperatures including frost, and consequently, HD is delayed for protecting the shoot meristems to be damaged until the environmental conditions become favorable (Law and Worland 1997). Thermal time or growing degree day (GDD) estimated by different statistical models is the variable mostly used for predicting the timing in days for the transition from one phenological stage to the next (Allard et al. 2012; Cane et al. 2013).

Given this background, the presented study aimed to dissect the genetic regulation of flowering time and the detection of novel QTL and epistatic interactions underlying HD in winter wheat under different environments. The particular goals of the current study were to (1) accurately assess the interaction of flowering time with the environmental stimuli in a geographical context; (2) provide insights into environment dependent and independent genetic factors controlling HD, and (3) uncover genomic regions interacting epistatically to control flowering time in winter wheat.

## Material and methods

### Plant material

A broad genetic background of 213 elite bread wheat cultivars released between 1966 and 2016 was used. The set comprised cultivars from Germany (winter type that needs vernalization and requires long days to start flowering), and other Western European countries, as well as cultivars from Mexico, India, USA, Australia, Moldava, and Chile (winter and facultative types) (Voss-Fels et al. 2019). We used two panels for the genome-wide association studies (GWAS). Panel1 included the 162 cultivars developed and adapted in Germany. Panel2 groups all the 213 cultivars of the set including the cultivars of panel1 plus the non-adapted cultivars originating from outside Germany.

### Experimental set-up

The experiments were conducted using an alpha design in three consecutive years from 2015 to 2017 at six locations across Germany: Moosburg an der Isar 48°28′N/11°56′E (Loc1), Klein-Altendorf 50°37’N /6°59´E (Loc2), Rauischholzhausen 50°46'N/8°53'E (Loc3), Quedlinburg 51°47'N/11°09'E (Loc4), Hannover 52°22'N/9°44'E (Loc5) and Kiel 54°19'N/10°08'E (Loc6). In total, 17 environments were included in the study (Loc3 was analyzed only in 2015 and 2016). More details of the experiment including plot size, seed rates, and drilling dates are described in Voss-Fels et al. (2019), whereas meteorological records are given in Table S1.

### Scoring of heading date and measurements of environmental factors

**Fig. 1:**
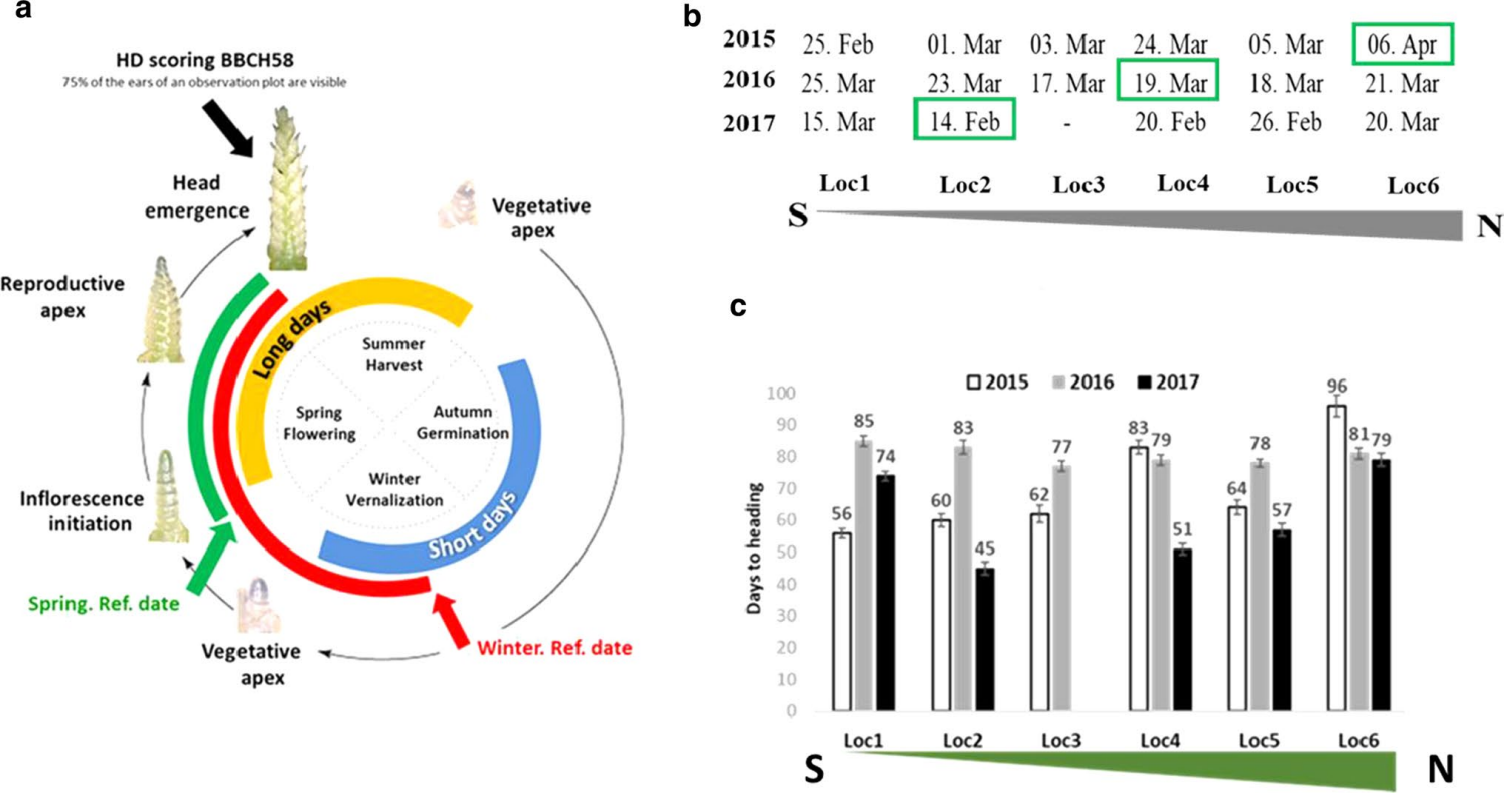
HD scoring based on winter and spring reference dates. **a**: Schema showing the seasonal control of heading in temperate wheat (winter type). After sowing in autumn, the plant vernalizes and the vegetative apex is growing slowly over winter and short days. Flowering time is delayed to protect the floral organ to be damaged because of cold (frost). When the days lengthen in spring, the vegetative apex transits into the reproductive apex, which indicates the inflorescence initiation as a response to favorite conditions of ambient temperature and long days. **b**: Reference dates corresponding to the first day from which growing degree day (GDD) kept being positive until the day of reaching the heading stage BBCH58 in each location*year. Abbreviations: Feb: February, Mar: March, Apr: April. **c**: The cold periods (in days) calculated from 1st January until the first day from which growing degree day (GDD) kept being positive until the day of reaching the heading stage BBCH58 in each location*year

HD was recorded according to two reference dates: the first one (HD_winter), as the number of days from January 1^st^ until the day when 75% of the ears of an observation plot were visible according to stage BBCH58 (Biologische Bundesanstalt, Bundessortenamt und Chemische Industrie) (Meier 1997). The second one (HD_spring) was recorded from the first day where GDD kept being positive for at least five consecutive days until the day of reaching the BBCH58 stage (day of heading) in each environment as shown in Fig. 1a. The accumulated GDD is calculated using the Peterson equation (Peterson 1965): $$\mathop {GDD = \sum\nolimits_{i = 1}^{n} \begin{gathered} \left\{ {\left( {\left. {\frac{T\max + T\min }{2}} \right) - Tb} \right.} \right\} \hfill \\ \hfill \\ \end{gathered} }\limits_{{}}^{{}}$$, where *n* = the number of days taken for the completion of a particular growth phase. In this experiment, the n represents the scored number of days to HD. The basic threshold temperature used for wheat is (Tb) = 4.0 °C (Cao and Moss 1989). HD_spring reflects the real number of days needed to complete the phenological stage “heading” based on the positive accumulated GDD after the cold period, which varied from year to year in the same location and between locations in a year (Fig. 1b, 1c). The measurements of the environmental stimuli were recorded beginning from both reference dates until the day of heading. The daily measurements of temperatures, global solar radiation, and precipitations were obtained from local weather stations placed directly at the experimental field in each location (Table S1). For temperature, the maximal (Tmax) and minimal (Tmin) values were calculated from the reference date until the day of heading for a given cultivar. For the other factors, the accumulated values of daily measurements starting from the reference date until the day of heading were used. Day length, including civil twilight (*h*), was computed daily following Forsythe et al. (1995).

Field trials were conducted in plots of size between 4.5 and 12 m^2^. The experimental sites had diverse soil characteristics, and sowing density was 330 viable seeds per m^2^ in 2 replicates.

### Allelic variation analysis of flowering time known genes

All cultivars were screened for known vernalization (*VNR1, VRN2, and VRN3*) and photoperiod (*PPD1*) genes. The genotyping included the recessive and dominant alleles of *VRN-A1*(*vrn-A1*, *Vrn-A1a, Vrn-A1b, Vrn-A1c)* (Yan et al. 2004a)*, VRN-B1 (vrn-B1, Vrn-B1),* null alleles *ZCCT-A1, ZCCT-B1,* and *ZCCT-D1* (Zhu et al. 2011), and functional alleles *ZCCT-A2, ZCCT-B2* and *ZCCT-D2* of *VRN2* (Distelfeld et al. 2009; Kippes et al. 2016)*,* photoperiod-insensitive alleles *Ppd-A1a, Ppd-B1a, Ppd-D1a* and sensitive alleles *Ppd-A1b, Ppd-B1b* and *Ppd-D1b* of *Ppd1* (Beales et al. 2007; Nishida et al. 2013)*.* The primers and the protocols used to amplify the target fragments are summarized in Table S2. DNA extraction was conducted following the protocol of DNAeasy Plant Mini Kit (Qiagen, Hilden, Germany). The polymerase chain reactions (PCR) were performed in a 25 μL reaction volume containing 100 ng of genomic DNA, 1 × Taq DNA polymerase reaction buffer, 10 μM of each forward and reverse primer, 0.2 mM of dNTP, and 0.5 unit of Taq DNA polymerase (NEB, Frankfurt, Germany). The PCRs were conducted in the thermocycler Flex cycler (Analytik GmbH, Jena, Germany). PCR profiles were visualized by electrophoresis on a 1% agarose gel stained with 0.04 μl/mL peqGreen (VWR, Darmstadt, Germany).

### Statistical data analysis

Analysis of variance (ANOVA) was performed adopting the general linear model (Gilmour et al. 1995) in the Proc Mixed procedure in SAS 9.4 (SAS Institute 2015). Variance components of genotypes (*G*), locations (L), years (*Y*) as well as their interactions (G ^*x*^ Y), (G ^*x*^ L), and (G ^*x*^ L ^*x*^ Y) were determined by the restricted maximum likelihood (REML) method assuming a random model in SAS 9.4. Broad-sense heritability (*H*^2^) estimation was calculated following the method described by Holland et al. (2003) $H^{2} = \frac{{V_{G} }}{{V_{G} + \frac{{V_{GxE} }}{E} + \frac{{V_{E} }}{E}}}$ where ${V}_{G}$: genetic variance, *V*_*G*_^*x*^_*E*_: variance of genotype × environment,$E$: environment, ${V}_{E}$: variance of the error term. Principal component analysis (PCA) for the phenotypic raw data was run using the function prcomp built-in *R* (Team 2013). Calculation of Pearson coefficients of the correlation and the partial correlation was performed in *R* using “cor” and “pcor” functions (Kim 2015).

### Genome-wide scan for marker-trait associations

The diversity panel was genotyped using the map of 24,216 informative SNP markers based on Infinium iSelect 15 K chip and the 135 K Axiom Exome Capture Array (Dadshani et al. 2021). Principal component analysis for the genotypic data was performed by using the prcomp core function in *R* (Team 2013). Marker-based identity-by-state (IBS) kinship matrix was calculated with the “A.mat” function of the *R* package rrBLUP (Endelman 2011) and the Pair-wise measures of linkage disequilibrium (LD) between two SNP with the package PLINK version 1.9 (Chang et al. 2015).

Genome-wide association mapping was done using the PROC MIXED procedure in SAS 9.4. For that the GRAMMAR method described by Aulchenko et al. (2007) was adopted. The first three principal components and the kinship matrix were included as co-factors to control for population structure. GWAS was conducted following the linear model: *Y*_*ik*_ = *µ* + *M*_*i*_ + *E*_*k*_ + *M*_*i*_^*x*^ *E*_*k*_ + *ε*_*ijk*,_ where $Y_{ik}$ is the vector of phenotypic values, which have been adjusted according to Aulchenko et al. (2007); *µ*: general mean, ${M}_{\mathrm{i}}$: the fixed effect of *i*-th marker; ${\mathrm{E}}_{\mathrm{k}}$: the fixed effect of the *k*-th environment (location-by-year); ${\mathrm{M}}_{\mathrm{i }}x{ E}_{\mathrm{k}}$: the fixed interaction effect of the *i*-th marker with the *k*-th environment, and ${\varepsilon }_{\mathrm{ijk}}$: the residual. Further increase in accuracy for detection of true QTL was achieved by the implementation of a tenfold cross-validation procedure with 20% leave-outs. The conditional analysis was included to check the peak marker behavior toward the surrounding neighbor markers in a region of 10 million bp. The forward selection and backward elimination approaches described in (Bauer et al. 2009) were implemented to validate the true QTL. The LOD threshold was determined based on false discovery rate (FDR) test of Benjamini and Hochberg (1995). For each GWAS (Manhattan plot), the FDR was set at 5% as suggested by Benjamini & Yekuteli, (2005) and Doerge and Churchill (1995) in the QTL model (Kilpikari and Sillanpää 2003). The genetic variance explained by a single SNP marker (*P*^*G*^) was calculated as follows: *P*^*G*^ = SQ_M_/SQ_g_, where SQ_M_ is the sum of squares of _i_th_ marker and SQ_g_ was calculated as the type I sum of squares (Type I SS) of the genotype in the ANOVA model. The total proportion of the genotypic variance *P*_*G*_ for each marker was calculated by including all markers with QTL effect in the ANOVA model. Since we used environment as a fixed factor, the conclusions of this study are limited to the tested environments.

### Identification of epistatic interactions

The two-way multilocus approach was used for epistatic interactions performed in a PROC MIXED procedure in SAS 9.4. In order to reduce the computational load, only markers that show a lower *p*-value of 0.7 (*P* < 0.7) were included in the analysis. The rationale behind this is, that the additive by additive epistatic variance is part of the additive genetic variance, which is measured in a GWAS. Markers that do not show any of these combined additive genetic and additive by additive epistatic effects probably do not possess any additive or any additive by additive epistatic effect. Thus, the number of the tested marker by marker combinations was drastically reduced. The model involved the environment factors as follows: *Y*_*ijk*_ = *μ* + *M*_*1i*_ + *M*_*2j*_ + *M*_*1i*_ × *M*_*2j*_ + ${E}_{k}$+*M*_*1i*_ × *M*_*2j*_ × ${E}_{k}$+*ε*_*ijk*_, where *Y*_ijk_: the vector of phenotypic values; μ: general mean; *M*_1i_: the fixed effect of *i*-th marker1, *M*_2j_: the fixed effect of *j*-th marker2, *M*_1i_ × *M*_2j_: the fixed interaction effect of *i*-th marker1 with *j*-th marker2_,_ ${E}_{\mathrm{k }}:$ fixed effect of *k*-th environment (location-by-year), *M*_1i_ × *M*_2j_×${E}_{\mathrm{k }}:$ fixed interaction of the *i*-th marker1 with the *j*-th marker2 genotype and *k*-th environment; *ε*_ijk_: the residual. Thresholds of *P*-value ≤ 0.001 and FDR < 5% were implemented in the model for more accuracy in detecting true epistatic interactions. The proportion of the genotypic variance explained by every single epistatic interaction was calculated as the variance of interacting marker-loci (M1xM2) toward the total variance.

### In silico DNA sequence analysis

The known vernalization *VRN* and photoperiod *PPD* genes were mapped physically on the wheat genome sequence RefSeqv2.1 (Zhu et al. (2021), https://urgi.versailles.inrae.fr/download/iwgsc/IWGSC_RefSeq_Assemblies/v2.1/) using the following approach: the core sequence of the markers (the specific sequence surrounding the SNP) was blasted against the genome sequence draft (Table S3). Further, the genes included in the flanking regions were downloaded and their annotations were checked using the last updated version of the gene annotation from the International Wheat Genome Sequencing Consortium and EnsemblPlants platforms. The start position of each gene was extracted from blasting outputs and was used later in the QTL and epistatic analyses. For some reported SSR markers, only the primer sequences were available in the GrainGenes database (www.wheat.pw.usda.gov). In this case, the sequence of the primers was blasted to find the corresponding physical positions, and the same steps were followed for blasting using the IWGSC RefSeq v2.1 gene annotation platform (Zhu et al. 2021).

The flanking region of each QTL is indicated in Table 3 using the ± 1LOD score to delimitate the QTL confidence intervals.

## Results

### Phenotypic assessment of heading date-by-environment interactions

**Fig. 2:**
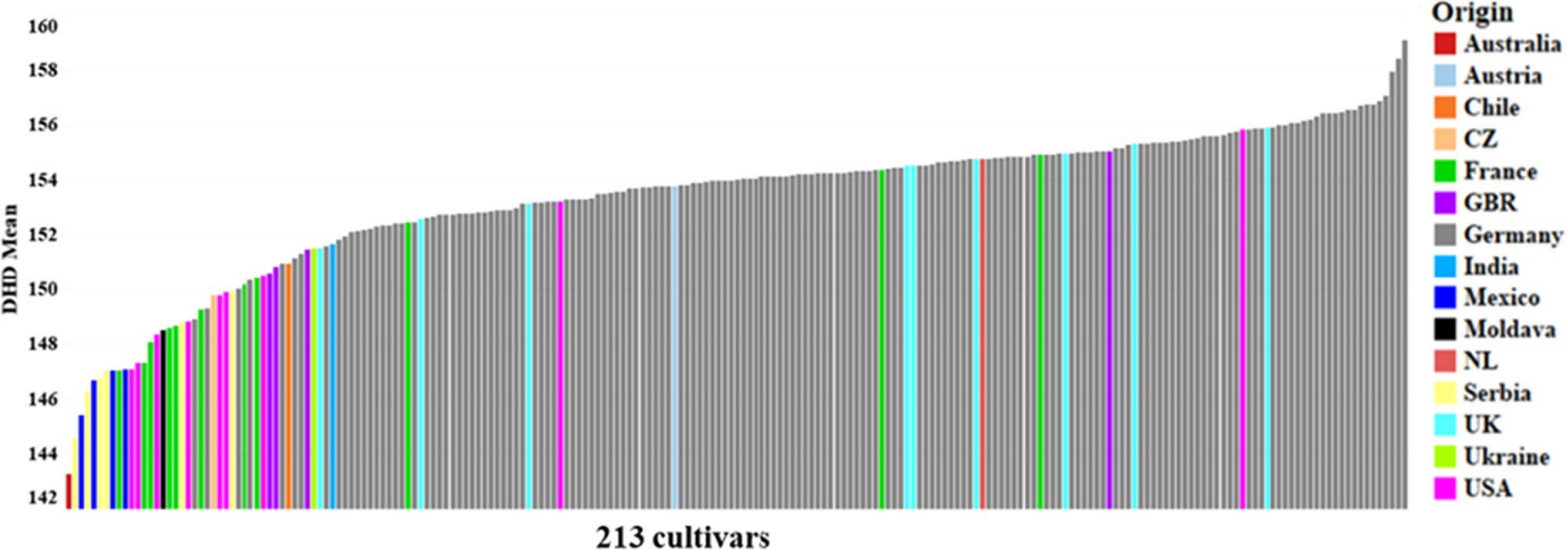
Phenotypic distribution of HD_winter in mean value per country of origin of 213 cultivars of the diversity wheat panel (panel2). The mean is based on data collected from six locations across Germany and over 3 years 2015, 2016, and 2017

To evaluate realized phenotypes, the genotypes of panel1 and panel2 were tested in six different locations for 3 years. The mean HD_winter across all environments was 10.4 and 16.2 days for panel1 and panel2, respectively (Table 1). The variance components of genotype and interactions genotype-by-year, genotype-by-location, and genotype-by-location-by-year were higher in panel2 compared to panel1. The heritability estimation was high, 0.89 for adapted cultivars, and 0.96 including exotic ones. The cultivars originating from Australia, Mexico, Serbia, Moldova, and the USA were found in the early flowering group (Fig. 2). Cultivars from France were the earliest flowering ones in the European germplasm. All latest flowering cultivars originate from Germany.

**Table 1:** Summary statistics for heading date for panel1 and 2

	Panel1	Panel2
Max	159.32	159.32
Min	148.92	143.12
Mean	154.12	151.22
SD	6.03	6.36
CV	3.93	4.18
σ^2^_G_	1.13***	2.54***
σ^2^_G *x* Y_	2.14***	3.04***
σ^2^_G *x* L_	4.99***	6.87***
σ^2^_G *x* L *x* Y_	11.94***	14.37***
σ^2^_error_	2.52	2.51
H^2^	0.89	0.96

Standard deviation SD. Coefficient of variation CV (in percentage). Variance components for genotypic variance *(σ*^*2*^_*G*_*),* genotype-by-year variance (*σ*^*2*^_*G x Y*_), genotype-by-location variance (σ^2^_*G x L*_) genotype-by-location-by-year variance (*σ*^*2*^_*G x L x Y*_). ***Significance at < 0.001 probability level. Heritability *H*^2^

**Fig. 3:**
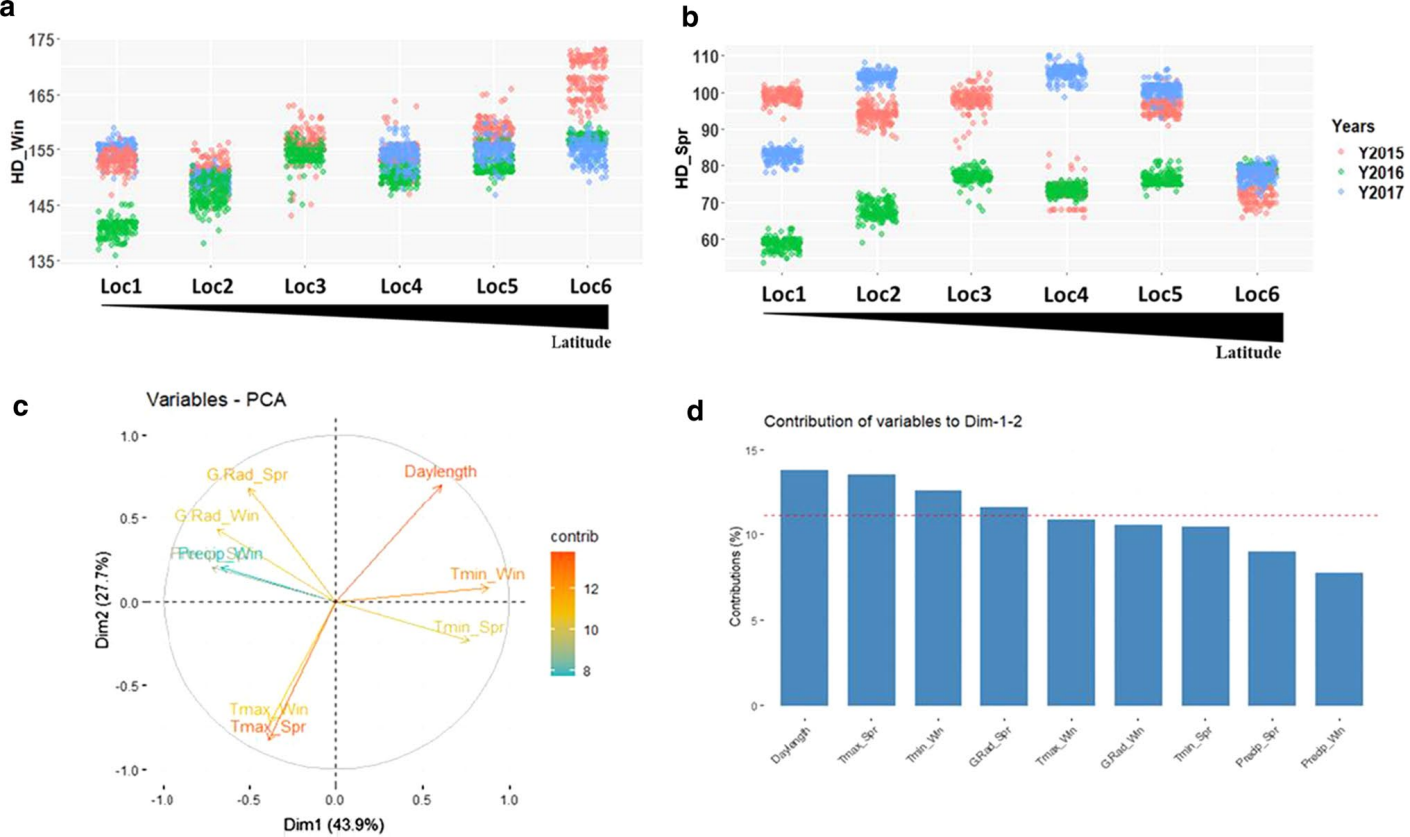
Comparison of HD variation based on winter and spring reference dates of scoring. **a** for HD-winter and **b**: for HD_Spring. Locations are denoted on the x-axes, HD scorings are denoted on the y-axes. The colors refer to years. **c**: Visualization of Principal Component Analysis of the variability among the environmental factors. The contributions of each environmental variable to the principal components Dim1 and Dim2 are indicated by percentage and colors. **d**: Summary of the contribution of each environmental variable by combining Dim1 and Dim2. The red dashed line indicates the expected average contribution. The environmental factors that are below the red threshold of the expected average contribution are considered less important

For a precise estimation of the environment effect, HD was evaluated using winter and spring reference dates. HD_winter revealed less differences among environments due to the overlapping of the scorings in all locations over the 3 years. An exception is Loc6 (North), where HD was delayed by 14.5 days in 2015 compared to 2016 and 2017. HD in Loc1 (South) increased by 12.6 days in 2016 compared to the other years (Fig. 3a). For HD_spring, an overlapping of scorings over years was noticed only in Loc6, while in other locations, two to three distinguishable clusters could be differentiated. In 2016, we observed a reduction in days to heading in Loc1, Loc2, Loc3, and Loc5 by 54, 59, 68, and 72 days, respectively, except in Loc4 and Loc6 (Fig. 3b).

PCA was conducted to identify the combination of variables that better explained the environmental variability. The first two axes of the PCA accounted together for ca 72% (Fig. 3c). Day length, Tmax of spring, Tmin of winter, and global radiation of spring explained the total environment variability by 13.7%, 13.5%, 12.6%, and 11.6%, respectively (Fig. 3d).

An ANOVA was performed to check the genotype effect on HD variation in interaction with environmental factors that were previously selected through PCA (Table S4). The location influenced the HD variation due to the genotypic response to Tmax, day length, and global radiation by 53%, 34%, and 13%, respectively. The genetic response to the yearly change of Tmax (Figure S1) explained 70% of HD variation, while genotypic interactions with day length and global radiation were stable from year to year and led to very weak HD alterations.

### Effect of environment-induced genetic response on HD variation

To identify the effect of environment-associated climatic parameters on HD variation, correlation analysis in each location was performed. The correlation matrix of HD between sites and years showed a strong correlation between years in loc1 and loc2 and decreases in other locations for both reference dates (Figure S2a). Pearson coefficients using HD_spring values revealed a strong negative correlation between HD and Tmax. When Tmax is high, days to heading are reduced leading to early flowering in the South (*r* = − 0.99 in Loc1) but less so in the North (*r* = − 0.26 in Loc6) (Figure S2b, Table S5). This effect did not change if the global radiation (*r*´ = − 0.83 in Loc1, *r*´ = − 0.35 in Loc6) or Tmin (*r*´ = − 0.89 in Loc1, *r*´ = − 0.20 in Loc6) is considered as covariates. The impact of Tmin followed the same trend and showed a higher inducing HD effect with *r* = − 0.98, − 0.79, − 0.81, and 0.04 in Loc1, Loc2, Loc3, and Loc6, respectively, and was not influenced by Tmax and the global radiation. The global radiation correlates positively with HD in all locations, except in the higher latitude (Loc6), where Tmax minimizes greatly the effect of the global radiation (*r* = 0.33, *r*´ = 0.85) on HD. Using the HD_winter scorings, all three factors showed a moderate and partial correlation with each other and with HD without a clear tendency to latitude. The correlation between HD and precipitations ranged from strongly positive to strongly negative for both reference dates depending highly on all other factors. Focusing on spring records, ANOVA revealed that the genotype response to Tmax changes explained 98.4% and 10.7% of HD variation in South and North, respectively, showing a strong reliance on latitude gradient. The genetic response to day length also depended on latitude but followed the opposite trend than Tmax. The interaction genotype-by-day length had a very weak effect on HD in the South and central regions, whereas increased to 89% in the North (from Loc4 to Loc 6). No significant HD changes could be explained by the genotype-by-radiation interactions in any location (Table 2).

**Table 2:** Percentage of the mean of squares extracted from ANOVA for the genotype interaction with environmental variables and heading date in panel1 (adapted germplasm) including six locations following latitude gradient

Source of variance	DF	Loc1 (South)		Loc2		Loc3		Loc4		Loc5		Loc6 (North)	
		MQ	%	MQ	%	MQ	%	MQ	%	MQ	%	MQ	%
Genotype*Tmax_Spr	161	788.43	98.4**	696.98	85.4**	184.67	96.4**	546.45	77.3**	40.35	10.5**	12.98	10.7**
Genotype*Daylength	161	9.41	1.2**	49.41	6.1**	6.89	3.6**	159.33	22.5**	293.34	76.3**	107.61	89**
Genotype*G.Rad_Spr	161	3.65	0.5**	69.31	8.5**	0.00	**0.00**	1.06	**0.00**	50.54	0.13**	0.23	**0.00**
Error		0.12		0.13		0.02		0.10		0.33		0.17	

Degree of freedom DF. Mean squares MQ. **Significance at the 0.01 probability level. Loc: Location. The maximal temperature of spring Tmax_Spr. Global radiation of spring G.Rad_Spr

### Genotyping the population for major flowering time regulatory genes

**Fig. 4:**
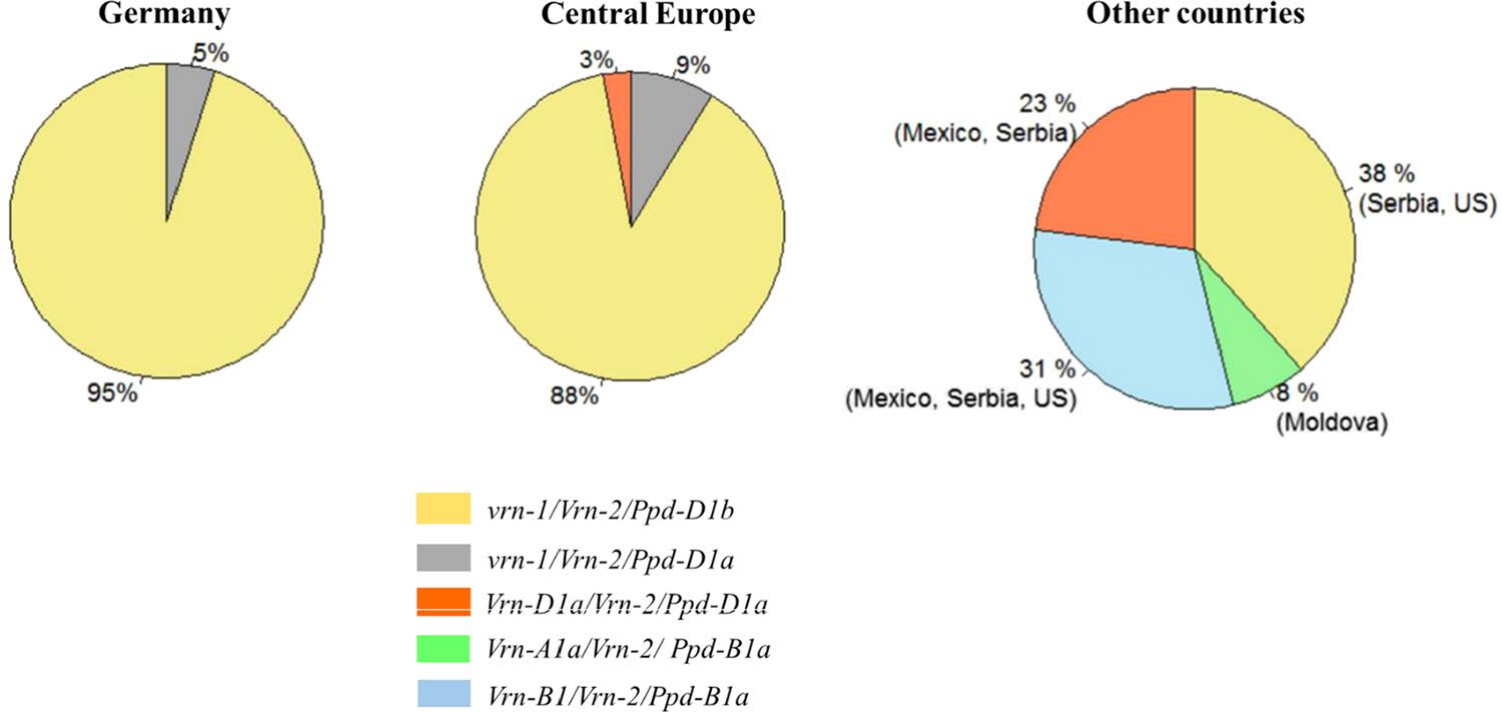
Frequency in the percentage of allele combinations of *VRN* and *PPD* genes detected in different wheat germplasms according to the country of origin. For vernalization genes, dominant and recessive alleles are designed with capital and small letters, respectively. For photoperiod genes, the letter “a” indicates the insensitive allele and the letter “b” the sensitive one

To identify the growth habit of the cultivars, they were genotyped for the known flowering time *VRN* and *PPD* loci (Table S6). For panel1, the analysis based on allele-specific primers using PCR revealed the presence of three recessive alleles *vrnA1, vrn-B1,* and *vrn-D1*, at locus *VRN1*. The screening of the *VRN2* loci on all three subgenomes showed the presence of null alleles *ZCCT-A1*, *ZCCT-D1,* and the absence of *ZCCT-B1,* as well the existence of the functional alleles *ZCCT-B2*, *ZCCT-D2,* and missing of *ZCCT-A2.* The photoperiod insensitive allele *Ppd-D1a,* and sensitive allele *Ppd-D1b* were also identified. In total, 95% of the adapted germplasm carry the allelic combination *vrn-1/Vrn-2/Vrn-3Bc/Ppd-D1b* (Fig. 4)*.* Only few cultivars (5%) harbor the insensitive allele *Ppd-D1a* beside the same *Vrn* alleles. For panel2, *Vrm-D1/ Ppd-D1* seemed to be the pair of alleles mostly associated with the growth habits of the European cultivars. Referring to the origin of selected cultivars, 88% of them from central Europe follow a winter growth habit. The facultative behavior related to *Vrn-D1a/Ppd-D1a* was detected in 9% of the cultivars from France and Serbia, while 3% of cultivars harbor *Vrn-D1a/Ppd-D1a*. Different allelic combinations that included mostly spring alleles (*Vrn-A1, Vrn-B1, Ppd-A1a,* and *Ppd-B1a)* were identified in the non-European wheat collection.

### Identification of environment dependent and independent QTL for heading date

We aimed to identify genetic regions controlling HD independently of environmental factors. For that, GWS including phenotypic data from all locations and years was performed. Based on this, setting criteria scores with LOD values higher than six was identified as QTL for panel1 and with higher than 15 for panel2.

**Fig. 5:**
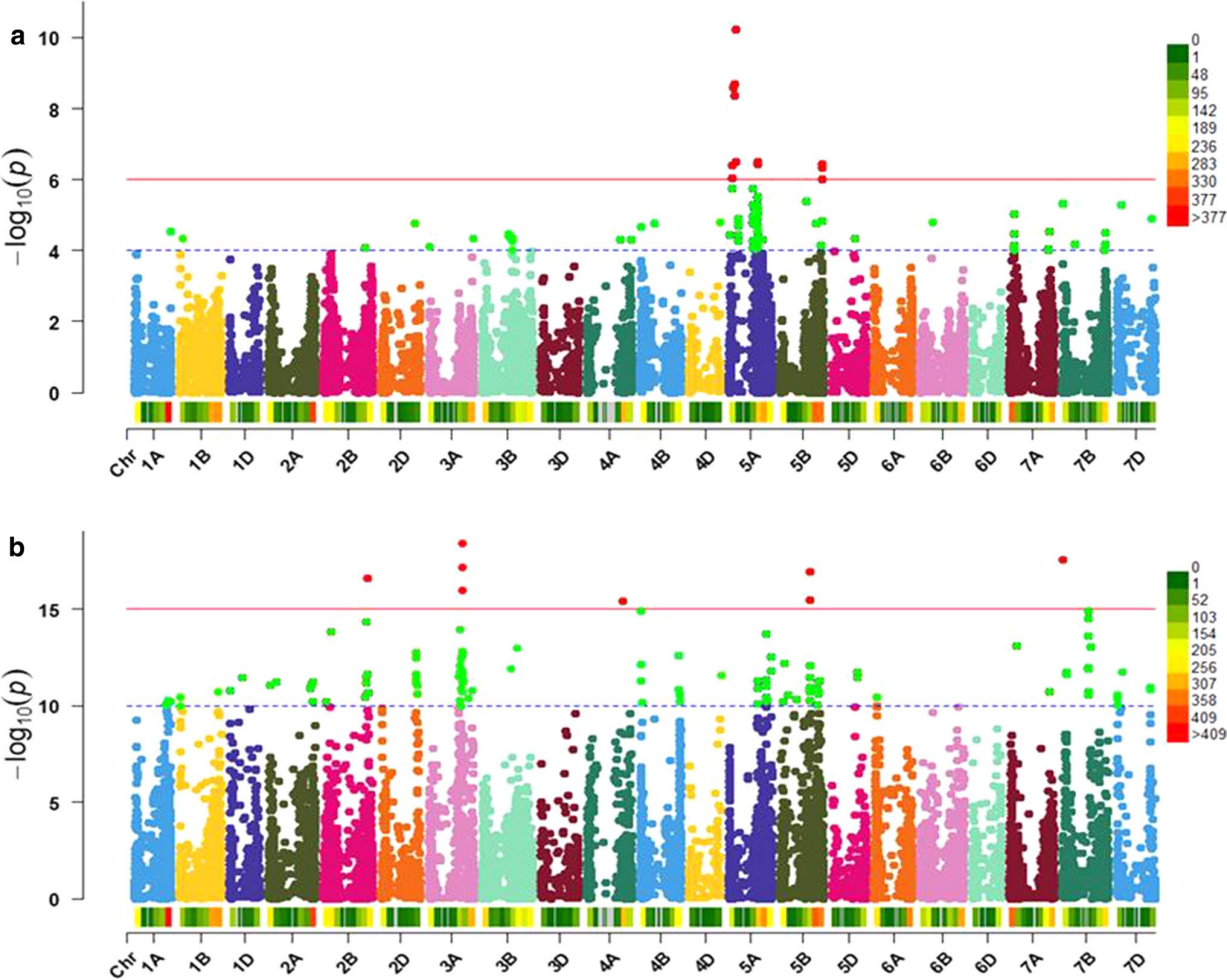
GWAS for heading date including phenotypic data from all locations and years using adapted (panel1) and adapted plus non-adapted (panel2) winter wheat cultivars. **a** and **b** Manhattan plot showing the identified QTL in panel1 and panel2, respectively. The y-axis refers to the –log10 (*P*) values of the SNP markers. The cut-off red lines indicate the genome-wide significance thresholds ( − log10 = 6, sb1) and ( − log10 = 15, sb2). The chromosomes are denoted on the x-axes. The red dots refer to the significant SNP markers above the cut-off red line. The SNP markers density per chromosome for each subset is shown above the x-axis. The number of SNP markers within 10 Mbp window size is indicated in categories and colors on the right side of the Manhattan plot

For panel1, four loci on chromosomes 5A and 5B were significantly associated with HD and consistent in different environments (Figure S3). The marker GENE_3500_336 mapped at 117,4 Mbp, explained the highest proportion of the genotypic variance (13.18%) with an allele effect of 1.2 days (Fig. 5a, Table 3). The detected QTL named TaHd102 is located in a region where no other QTL has been reported so far.

**Table 3:** Significant QTL for flowering time detected in the winter wheat association panels of panel1 and panel2

	QTL	Marker^a^	Chr^b^	Position^c^	Flanking region^d^	MAF^e^	F_Value^f^	*P*^g^	− Log_10_(*P*)	FDR^*h*^	*PG*^*i*^	Allele effect^j^	Present allele	RefSeqv2.1
Panel 1	TaHd098	Ra_c69221_1167	5A	41,427,451	36,273,096–51,590,002	0.37	26.06	9.40E–07	6.03	9.00E–04	2.78	0.97	T	T
	TaHd102	GENE_3500_336	5A	117,495,484	110,667,048–132,9633,164	0.47	49.3	6.14E–11	10.21	4.25E–07	13.18	− 1.2	T	T
	TaHd112	BS00022191_51	5A	476,402,782	461,485,853–481,199,152	0.35	28.54	3.14E–07	6.5	4.72E–04	2.46	1.05	T	C
	TaHd132	BS00024829_51	5B	693,611,551	691,411,951–697,289,998	0.26	28.11	3.75E–07	6.43	4.72E–04	2.21	− 1.19	T	T
Panel 2	TaHd034	AX-158603420	2B	720,796,133	720,796,133–730,190,623	0.11	120.4	2.45E–17	16.61	5.54E–19	1.54	5.09	A	C
	TaHd044	AX-111134276	3A	556,662,059	541,483,283–565,766,591	0.1	159.69	4.25E–19	18.37	1.97E–23	33.01	5.63	A	A
	TaHd072	AX-158581720	4A	593,486,064	581,869,248–596,506,881	0.12	113.35	3.86E–16	15.41	3.45E–18	1.77	6.27	A	G
	TaHd125	Jagger_c3991_101	5B	488,820,722	478,130,002–490,769,429	0.08	126.61	1.14E–17	16.94	8.06E–20	1.82	6.01	T	C
	TaHd171	AX-158601566	7B	2,944,225	1,980,522–3,500,643	0.09	155.01	2.68E–18	17.57	4.31E–23	7.09	5.83	A	G
Candidate genes in panel 2	*PPD-A1*	AX-158573607	2A	70,940,322	70,877,024–71,318,288	0.38	44.44	1.13E–11	10.95	7.97E–09	2.02	− 1.73	A	A
	*PPD-B1*	Exca_rep_c68899_1400	2B	91,836,538	89,552,942–93,545,484	0.14	71.37	8.15E–13	12.09	6.26E–13	0.04	1.66	A	A
	*VRN-D2*	RAC875_c8642_231	4D	509,666,717	498,241,876–512,102,050	0.08	91.32	1.12E–14	13.95	1.09E–15	2.02	1.55	T	C
	*VRN-A1*	AX-111486916	5A	587,411,454	586,141,645–588,872,113	0.11	17.12	5.07E–05	4.29	3.76E–04	1.21	− 1.98	A	G
	*VRN-A2*	BobWhite_c8266_227	5A	698,507,476	689,913,529–708,418,214	0.08	101.31	3.34E–15	14.48	6.18E–17	1.17	2.12	T	G

a The peak marker of QTL for flowering time showing the highest − Log10(*P*) b The chromosome harboring the peak marker. c The physical position in bp of the peak marker d The physical interval of the most significant QTL harboring the peak marker e The minor allele frequency set to > 5% f *F*-test statistic value *g P* value threshold set to *p* ≤ 0.001 h False discovery rate (FDR) set to ≤ 0.05 i Proportion of the genotypic variance explained by the QTL in %.^j^ Effect in days of the allele substitution on flowering time

By including the non-adapted cultivars in panel2, five QTL, different from the ones found in panel1 were identified on chromosomes 2B, 3A, 4A, 5B, and 7B (Fig. 5b, Table 3). These QTL were consistent in different environments (Figure S4). The peak marker AX-111134276 (at locus TaHd044) located at 556.60 Mbp on chromosome 3A had the strongest effect and explained 33% of the observed genetic variance. The allelic variation at this locus alters HD by 5.6 days.

No heading-date QTL was detected among the adapted cultivars for the five QTL loci identified in panel 2. No MTA related to *VRN* and *PPD* genes were detected in panel1, while associations to *VRN-A1*, *VRN-A2*, *VRN-B1*, *VRN-D2, PPD-A1,* and *PPD-B1* loci were identified in panel2 (Table 3). The detected MTAs related to known flowering time genes explained a lower proportion of the genetic variance compared to the QTL TaHd044 (Table S7). This QTL is located in a similar region where the QTL reported by Griffiths et al. (2009) and Zanke et al. (2014) is located. To check for possible overlapping of these loci, we compared the association data of the markers tagging these QTLs and of the surrounding ones in the panel used in this study. The genetic linkage analysis clearly showed that the two QTL, TaHd044 and WCM264 are located in chromosomal regions that segregate independently from each other (Figure S5). This finding was confirmed by the conditional analysis. Taken together we conclude that they likely have different underlying causal genes.

Further, for a better understanding of the genetic modulation of heading, we performed GWAS per each environment separately. In total, 95 SNPs distributed across 17 environments were identified (Table S8). Some shared QTL among the specific location-by-year combinations were detected. In 2015, three possibly homoeologous QTL (TaHd024, TaHd036, TaHd040) were uncovered at the very distal end of chromosomes 2A, 2B, and 2D, respectively. This region was shared by locations at lower latitudes until the middle part of Germany (Loc1 to Loc3), whereas northern regions (Loc5 and Loc6) had a common QTL (TaHd122) on the short arm of chromosome 5A. The year 2016 was the warmest among the 3 years of the experiment in the southern and central locations (Loc1 to Loc3) that shared the loci TaHd059 and TaHd088 on chromosomes 3B and 4B, respectively. The loci detected in 2017 followed no trend with latitude gradient. The overall effect of revealed environment specific QTL spans from inducing early flowering time by 2.6 days (Loc5 in 2016) to delaying it by 4.45 days (Loc2 in 2015) (Table S8).

### Identification of epistatic interactions involved in heading date control in winter wheat

**Fig. 6:**
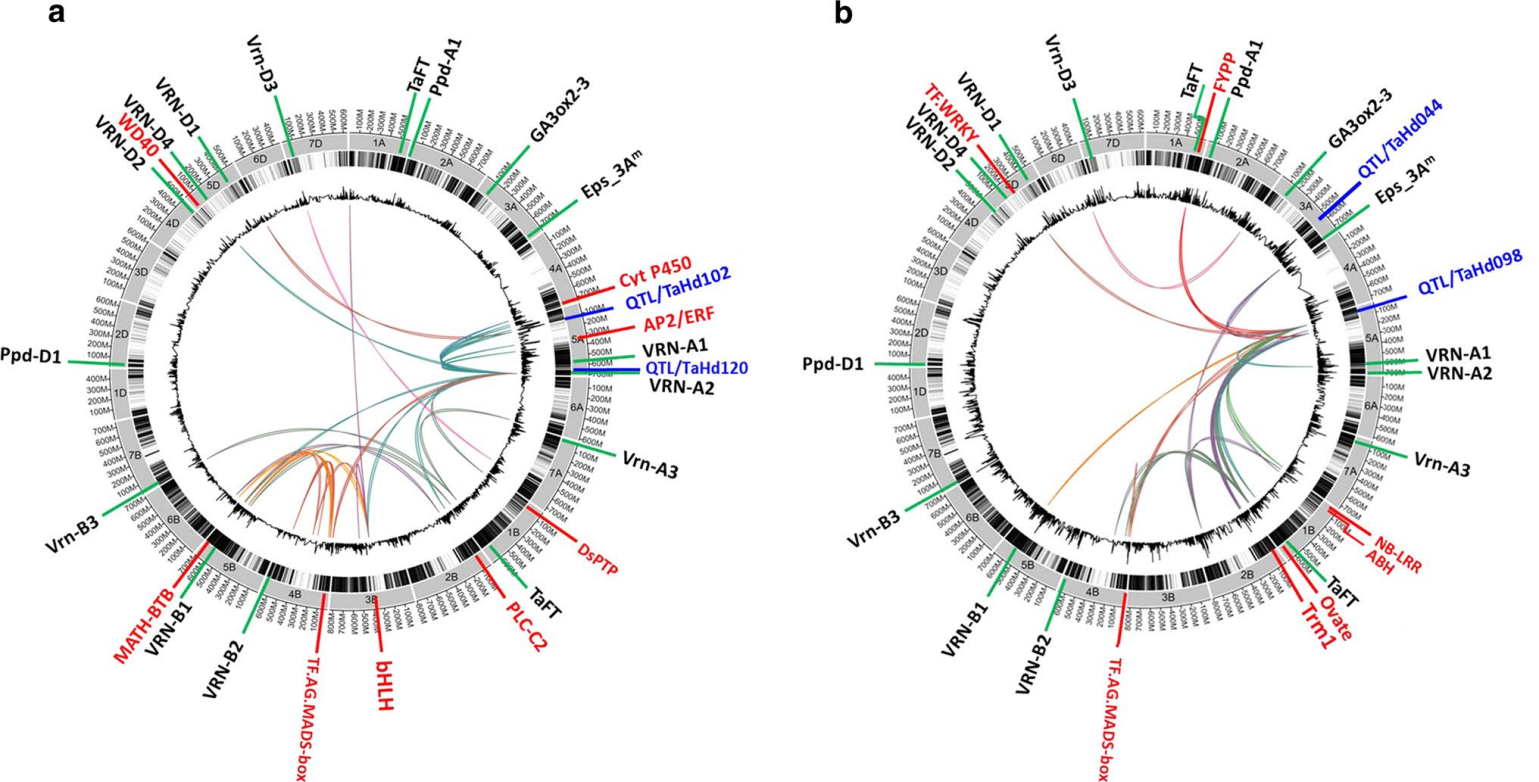
Epistatic interactions detected in panel1 **a** and panel2 **b**. From outside to inside, the layers indicate the length of chromosomes in Mb, then the organization of chromosomes per subgenome A, B, and D, then the mapping of SNP markers used for GWAS, then the QTLs presented according to their –log10 *P* values extracted from GWAS. The last inner curved lines indicate significant interactions between SNP markers highlighted in colors. The known flowering time genes are indicated with the green arrows. The detected genes are highlighted in red. The blue color designs the QTL with epistatic effect

To evaluate how the interaction among loci affects flowering time, genome-wide epistatic interaction analysis was performed. Using panel1, 32 significant epistatic interactions were detected explaining up to 3.8% of the genetic variance (Table S9). One locus on chromosome 5A (TaHd120) at 698.10 Mbp, 37 kb apart from the *VRN2* locus, was involved in 14 epistatic interactions with loci located on chromosomes 1B, 2B, 3B, 4A, 4B, 5A, 5B, and 5D including the strongest QTL TaHd102 identified in the same subset (Fig. 6a). We detected 30 significant epistatic interactions using panel2, which explained up to 7.8% of the genetic variance (Table S10). Two loci mapped on chromosomes 1B (TaHd015) and 5A (TaHd104) at 158.2 Mbp and 654.70 Mbp, respectively, showed the strongest epistatic interaction in the panel2, explaining 7.8% of the genetic variance. The combination of minor alleles of both regions induced HD by 4.64 days earlier compared with that of major alleles. The locus TaHd098 showing effect in panel1 was involved in 15 digenic interactions in panel2 (Fig. 6b).

## Discussion

### Response of heading date to local and seasonal interplays of environmental factors

In this study, a difference of 10.4 days was observed between HD of adapted cultivars within a latitude range of around 6°. HD variation between individuals across very small temporal and spatial scales was previously reported, as a resulting effect of the local climatic conditions that cause a part of within-population variation (Dahlgren et al. 2007). The reduced genotypic variance of HD in panel1 compared to panel2 is attributed to the impact of local adaptation of the German cultivars. In the environments analyzed in this study, the genetic effect on HD variation was depending more on location than on year. This indicates the importance of multi-location trials with broad geographic distribution for the genetic estimation of a highly heritable trait such as HD (Holland et al. 2003). Moreover, the high variance of genotype-by-location-by-year interaction for both sets revealed that all cultivars respond differently to the 17 environments. This shows that the used germplasm has a high genetic potential.

The interplay of climatic factors affects all phenological events of plants including flowering time in barley (Jones & Thornton 2003), rice (Prasad et al. 2006), and wheat (Kouchaki & Nasiri 2008). Exploiting GDD as an indicator of growth revealed the dominant effect of Tmax and Tmin of spring toward other factors in reducing days to heading from the lowest latitude to the middle ones. The HD inducing effect of temperature was reported by other studies (Menzel et al. 2006; Miller-Rushing et al. 2007; Record 2009; Moore & Lauenroth 2017). The elevated solar radiation accumulation was highly associated with delayed HD. High UV-B radiation plays a crucial regulatory role in plant growth and morphology (Bornman et al. 2015). However, some studies report the delay of flowering time as a response to high natural UV-B radiation in different plant species, such as maize (Saile‐Mark et al. 1996), roses (Terfa et al. 2014), and pea (Roro et al. 2016). The PCA showed that 72% of the environmental variation was explained by the parameters considered in the study. The remaining 28% could be due to other parameters such soil temperature and moisture.

### Temperature and day length affect flowering time in a latitude-dependent manner

**Fig. 7:**
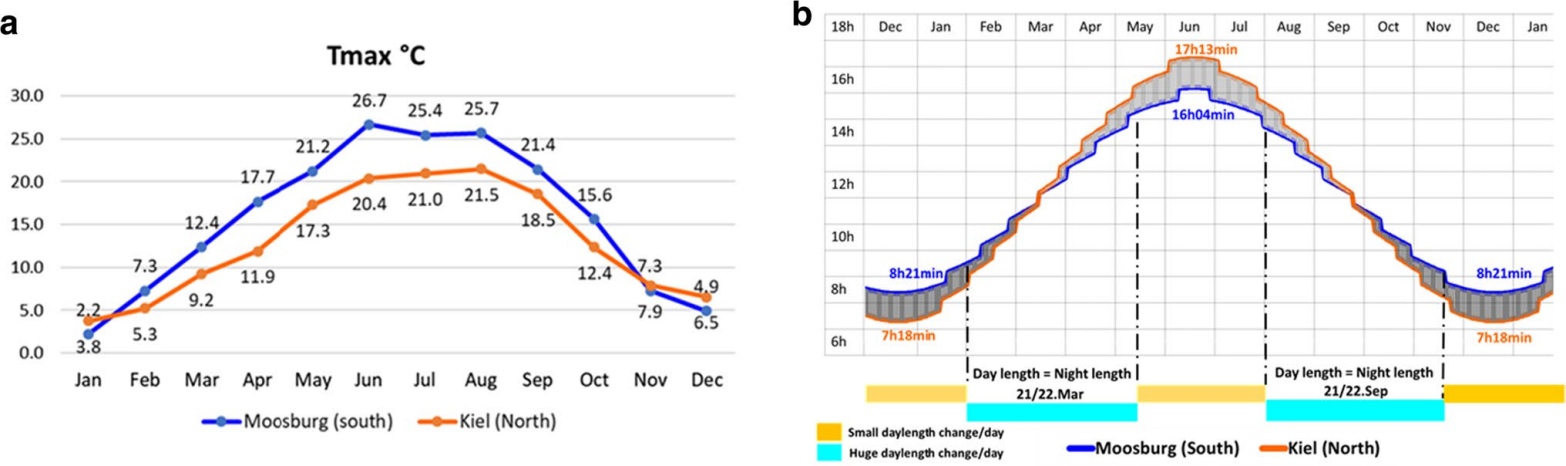
Seasonal change of Tmax **a** and day length **b** including 3 years in loc1 (Moosburg) and in loc6 (Kiel). The mean of Tmax per month is indicated in numbers. Day length, including civil twilight (*h*), was computed daily following Forsythe et al. (1995)

We did not observe a linear relationship between latitude and HD in the present study. On one hand, Villegas et al. (2016) reported that the long day length is more responsible for short “sowing to anthesis” duration than the temperature in a latitude range of 22°. On the other hand, the climatic stimulus that induces flowering time in one location is not necessarily the same in another (Wilczek et al. 2010). Both, Day length and Tmax of spring contributed mostly and quite equally in explaining the environmental variability. Therefore, the interactions between genotype, day length, and Tmax should be considered to understand the variation in HD associated with latitude. It is noteworthy that a dramatic acceleration of flowering with increasing light amount (as a result of day length) was observed in several annual plant species (Tsegay et al. 2005; Opseth et al. 2016; Chiang et al. 2018). The faster prolongation of day length during the spring season in the North than in the South, while Tmax recorded higher values (17–21 °C) in the South than in the North (11–17 °C) at the same period (Fig. 7), might explain the opposite genetic responses to these two environmental factors. Hence, the impact of high seasonal change of temperature in the South on HD seems to compete with the larger day length seasonal variation occurring in the North. Consequently, plants are adapted to use temperature as an indicator of favorable conditions in lower latitudes, whereas in the higher ones, they are using photoperiod as a more reliable proxy of the changing seasons for starting HD. Furthermore, because the annual thermal change is greater in the lower latitudes than in the higher ones, and as the seasonal alteration of day length is the only environmental factor that remains unchanged from year to year, might explain the constant HD behavior in the North and the increasing HD variation further South (Fig. 3b).

### The roles of *VRN* and *PPD* genes in flowering time control

The allele combination *vrn1/Vrn2* confers the winter growth habit. (Takahashi 1970; Yan et al. 2006). Indeed, the adapted germplasm carries the winter allele *vrn1* due to the three homoeologous recessive alleles *vrnA1, vrn-B1,* and *vrn-D1.* No triple recessive *ZCCT-1* combination was observed in panel 1 despite the detection of despite the detection of null alleles *ZCCT-A1* and *ZCCT-D1*. Nonetheless, the presence of functional alleles *ZCCT-B2* and/or *ZCCT-D2* leads to dominant *Vrn2,* and consequently to an increase in vernalization requirement (Distelfeld et al. 2009; Kippes et al. 2016). This result is in line with Langer et al. (2014), who reported that 82% of the European winter wheat cultivars harbor day length sensitive allele *Ppd-D1b* with 100% dominance of winter allele *vrn-1.* Since the majority of the adapted cultivars carry the same allelic variations at *VRN* genes, the HD range of 10.4 days shown by the German cultivars cannot be convincingly explained by the allelic variation at *PPD-D1* locus, as only 5% of the cultivars harbor the insensitive allele *Ppd-D1a.* The candidate gene approach revealed the presence of alleles at *VRN* and *PPD* loci established as a result of long-term adaptation to winter conditions. Nevertheless, the HD variation due to genetic effect and interaction with the environment very likely involves more genetic regulators responsible for HD variation. The *PPD1* alleles are classified as photoperiod sensitive (*Ppd1a*) or photoperiod insensitive (*Ppd1b*), with the latter heading earlier under SD and showing a reduced photoperiodic response. For the *PPD* locus, the insensitive alleles at *Ppd-A1* and *Ppd-B1* reported by Nishida et al. (2013) have an equal HD inducing effect as *PPD-D1*.

### Heading time QTL stable across environments

The overall effect (20.6%) of the four detected stable QTL in this study is higher compared to the six QTL (9.5%) reported by Langer et al. (2014) that tested more European winter wheat cultivars but in three close locations for one single year. Granted that the size of the population is a determinant factor in GWAS, the incorporation of the interaction with the environment may improve the power of GWAS to find QTL that are significant in a broad range of environments (Cantor et al. 2010; Thomas 2010). The TaHd102 associated with 13.15% of genetic variance on HD in panel1 is located distantly from the reported SSR marker *Xgwm293* in the small arm of chromosome 5A and is linked to a QTL for plant height reported previously (Griffiths et al. 2009) (Figure S6). We did not find other studies reporting so far QTL on heading in the TaHd102 region. Turuspekov et al. (2017) have detected one QTL on 5AS with a genetic positon of 11 cM. The TaHd102 produced significant effects over all studied environments suggesting that it can be used to adjust HD in different environments across the tested region. That no QTL related to *VRN* genes was detected in panel1 could be explained by the fact that all German cultivars are adapted to the same vernalization conditions as was also revealed by PCR screening. *PPD-A1* and *PPD-B1* loci do not harbor any polymorphic sites that segregate in the European germplasm as shown by Langer et al. (2014). Increasing the additive genetic variance of a trait in a mapping panel facilitates high-resolution mapping and allele mining (Ersoz et al. 2007; Uchiyama et al. 2013). Therefore, the incorporation of the non-adapted cultivars enabled uncovering the strongest MTA at the locus TaHd044. The latter is flanked by two previously reported SSR markers Xbarc45 (Griffiths et al. 2009) and WMC264 (Zanke et al. 2014), but as shown they are located at different positions in the genome being far apart from TaHd044. Similarly, in a quite different part of chromosome 3A is the QTL reported by Martinez et al. (2021). Nevertheless, the QTL on 3AL that might overlap with the interval of TaHd044 is detected by Luján Basile et al. (2019). Further studies using linkage biparental populations are needed to determine if they are different or the same QTL. The identification of loci related to *VRN* genes in panel2 is most probably due to different vernalization requirements, caused by the non-adapted alleles represented mainly in non-German germplasm, which could carry natural variations that lead to a need for shorter exposure to cold (Yan et al. 2004a; Fu et al. 2005; Kippes et al. 2015). The detected alleles can be introgressed into the adapted breeding wheat cultivars of improved adaptability to face the challenging climate changes.

### Environment specific QTL are affected by climatic parameters

The heading time QTL detected in the studied environments are likely affected by latitudinal-dependent environmental cues. They can be exploited to enhance adaptability to different environments. The three QTL, uncovered in the lower latitudes, TaHd036, TaHd059, and TaHd088, are located in regions that include homologs related to genes involved in the response to ambient temperature in Arabidopsis, heat-inducing control of spikelets number in rice, and thermotolerance regulation in tomato, respectively (Liu et al. 2012a, b; Wang et al. 2019; Wu et al. 2017). The TaHd122 QTL region includes a gene belonging to the Auxin/B3 gene family and was significant only in the higher latitudes, where the photoperiod acts as a reliable proxy for initiating the floral transition.

### Epistatic interactions

The locus TaHd120 located in the *VRN2* locus region is implicated in 14 genetic interactions. This strongly suggests that *VRN-A2* plays a central role in the regulatory network controlling heading time in the adapted germplasm. The epistatic effect of *VRN* loci in the genetic control of flowering time in the European winter wheat was reported by Reif et al. (2011) who found that the *VRN-A1* gene is involved in four epistatic interactions. The identification of ORFs in the intervals interacting with *VRN2* revealed the *Apetala2/Ethylene (AP2/ERF)* on chromosome 5A, class of genes that are well described in regulating the correct timing of the transition of the spikelet meristem to the floral meristem in maize (Chuck et al. 1998). Similarly, we found that the other chromosomal regions interacting with the *VRN2* harbor genes for protein families such as *MATH-BTB, bHLH, WD40, Agamous/MADSbox, DsPTP1, and PLC-C2*, known as regulators of flowering time in other plant species (Hazebroek and Metzger 1990; Yanofsky et al. 1990; Sheldon et al. 1999; Georges et al. 2009; Ito et al. 2012; Chen et al. 2015; Jiang et al. 2018). Interestingly, TaHd098 that is less significantly associated with HD in the adapted germplasm, showed a strong epistatic effect when adding the non-adapted cultivars to the analysis. Some of the 15 interacting loci were mapped very close to key regulatory elements of flowering time in *Arabidopsis* like *FYPP* (Kim et al. 2002), *Alpha–Beta hydrolase (ABH)* (Sun and Ni 2011), and *tRNA methyltransferase (Trm1)* (Guo et al. 2019) on chromosomes 1A, 1B and 2B, respectively. TaHd098 interacts with known HD genes in wheat such as *TaFT3, Eps-3A, VRN-B1,* and *Vrn-3/FT* genes on chromosomes 1A, 1B, 3A, 5B, and 7A, respectively.

## Conclusion

In this study, we obtained insights into aspects of the complex interaction of the environmental factors with flowering time regulation in wheat. The impact on HD of high seasonal changes of temperature in the lower latitudes competes with great day length seasonal variations occurring in the higher ones in the period from winter to spring. The resulted genetic response selects thermo-sensitive loci in the South and photoperiod susceptible loci in the northern locations for starting the transition to the reproductive phase. The allele combinations of *VRN* and *PPD* genes responsible for the winter and facultative growth habits of adapted and non-German cultivars were determined. We enriched our understanding of the flowering time pathway in wheat with one QTL TaHd102 on chromosome 5A attributing a consistent effect across multiple environments, and another allele TaHd044 on chromosome 3A not frequent in German cultivars that induces greater HD alteration and have not been reported so far. In addition, tuning QTL that respond to specific environmental stimuli were identified. The locus TaHd098, detected on chromosome 5A, is implicated in more epistatic interactions for controlling flowering time in non-adapted winter wheat. Further, we propose a pivotal epistatic role of *VRN2* on HD based on its multiple genetic interactions with key regulatory elements in the adapted germplasm. Our findings offer new insights into understanding the mechanisms of the genetic architecture underlying flowering time in winter wheat and can be leveraged for the wheat breeding process to develop cultivars adapted to different environments. Further genetic and molecular analyses are needed to conclusively prove that the here reported QTLs are unique and that they have other underlying causative genes. Considering the strong environment independent effect of this allele on HD, it can be exploited to breed new cultivars with improved adaptability to future climatic conditions.

## Supplementary Information

Below is the link to the electronic supplementary material.

**Figure S1** Boxplots showing the measurements of climatic factors per environment according to winter and spring reference dates. Each boxplot in each measurements is based on the scorings per genotype. The mean was considered for the comparison between environments. a, b) The maximal temperature in °C. c, d) The minimal temperature in °C. e, f)The accumulative global radiation in Mj/m2/day. g, h) The accumulative precipitations in mm. i) The daylength in hours(PDF 290 KB)

**FigureS2a** Correlation matrix showing Peasron correlation coefficients between 17 environments using heading scores based on winter (HD_Win) and spring (HD_Spr) reference dates (PDF 68 KB)

**Figure S2b** Geographical heatmap summarizing the correlation between the climatic factors and HD (PDF 68 KB)

**Figure S3** Manhattan plots showing the identified QTL for heading date per environment in panel1 (PDF 1089 KB)

**Figure S4** Manhattan plots showing the identified QTL for heading date per environment in panel2. (PDF 351 KB)

**Figure S5** Manhattan plot of genome-wide association study (GWAS) zooming the chromosome 3A interval harboring the TaHd044 QTL. The red dots and arrows indicate the SNP AX-111134276 that detected TaHd044 and the nearest SNP AX-158533114 to SSR WMC264 (Zanke et al.2014). The color scale on the left shows the strength of LD, from blue the weakest to red the strongest. X-axis: the position of the markers in base pairs and y-axis: the LOD values as –log10(p) (PDF 156 KB)

**Figure S6** Physical mapping of strongest detected QTL for heading date trait using panel1 (marker in red color) and panel2 (marked in green color) on chromosomes 5A and 3A, respectively (PDF 40 KB)

**Table S1** Summary of heading date scorings and daily measurements of environmental factors per location and year. Table S2 Primer used for the analysis of allelic variation at VRN and PPD genes. Table S3 Physical mapping of VRN and PPD genes based on reported flanking Marker in cM. Table S4 ANOVA of climatic variable and heading date depending on location and year. Table S5 Pearson coefficients of correlations between HD and the environmental parameters . Table S6 Genotyping of 213 cultivars at known VRN and PPD genes via PCR. (+) and (-) indicate the presence and absence of the corresponding alleles. Table S7 Genotypic variance of QTL TaHd044 compared to VRN and PPD genes. Table S8 Environment specific QTL per location and year in panel1. Table S9 Epistatic interactions detected in panel1. Table S10 Epistatic interactions detected in panel2 (XLSX 106 KB)

## Data Availability

The datasets generated during and/or analyzed during the current study are included in this manuscript and supplementary material. All the raw data are available from the corresponding author on reasonable request.
