# Correction to: Identification of QTLs for wheat heading time across multiple‑environments

**DOI:** 10.1007/s00122-022-04188-8

**Published:** 2022-09-17

**Authors:** Salma Benaouda, Said Dadshani, Patrice Koua, Jens Léon, Agim Ballvora

**Affiliations:** 1 Institute for Crop Science and Resource Conservation, Chair of Plant Breeding, Rheinische Friedrich-Wilhelms-University, Katzenburgweg 5, 53115 Bonn, Germany; 2 Rheinische Friedrich-Wilhelms-University, Bonn, Germany

## Correction to: Theoretical and Applied Genetics (2022) 135:2833–2848 https://doi.org/10.1007/s00122-022-04152-6

The legends of supplementary figures and tables have been missed out in the original publication.

The original article has been corrected.

