# Supplementary figures and images for: Identification of QTLs for wheat heading time across multiple-environments
Source: Theor Appl Genet. 2022 Jul 1;135(8):2833–48. doi: 10.1007/s00122-022-04152-6 (PMC9325850; doi:10.1007/s00122-022-04152-6)

**a**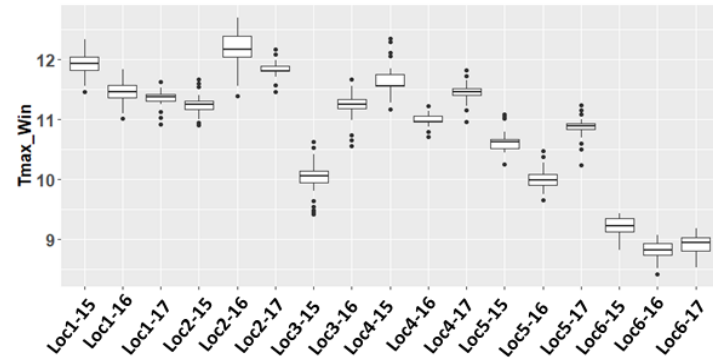**b**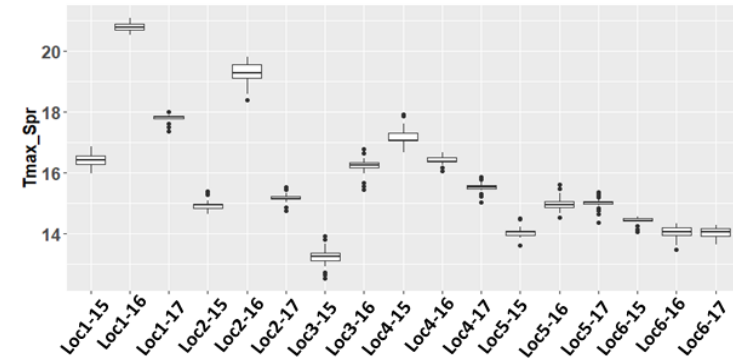**c**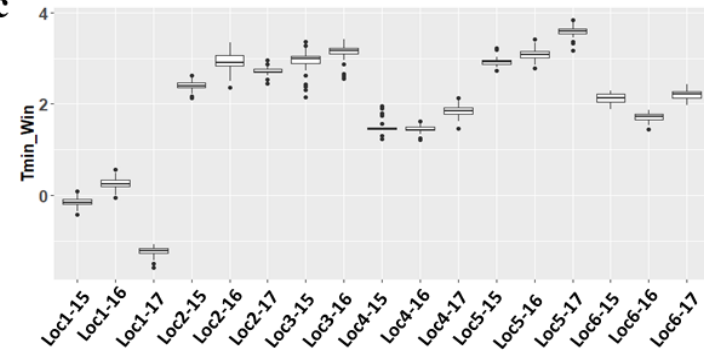**d**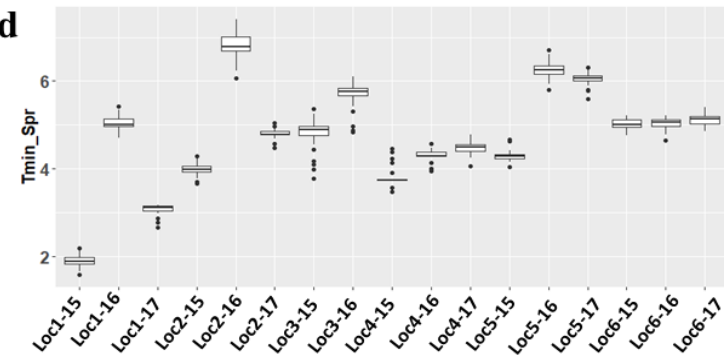**e**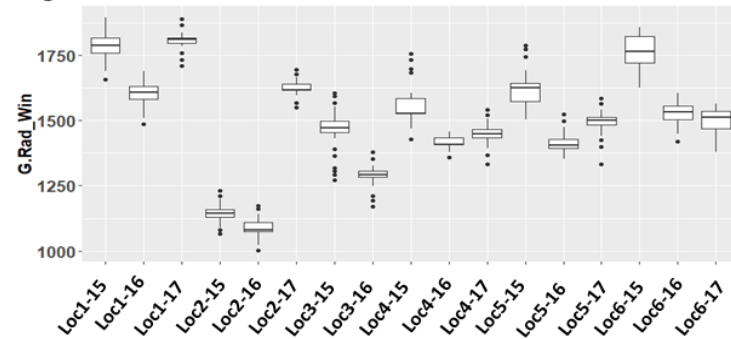**f**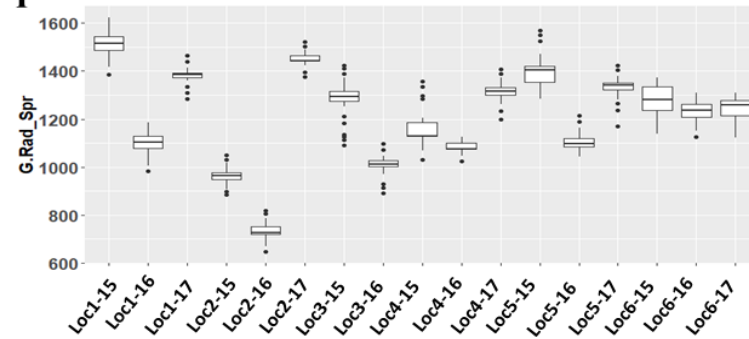

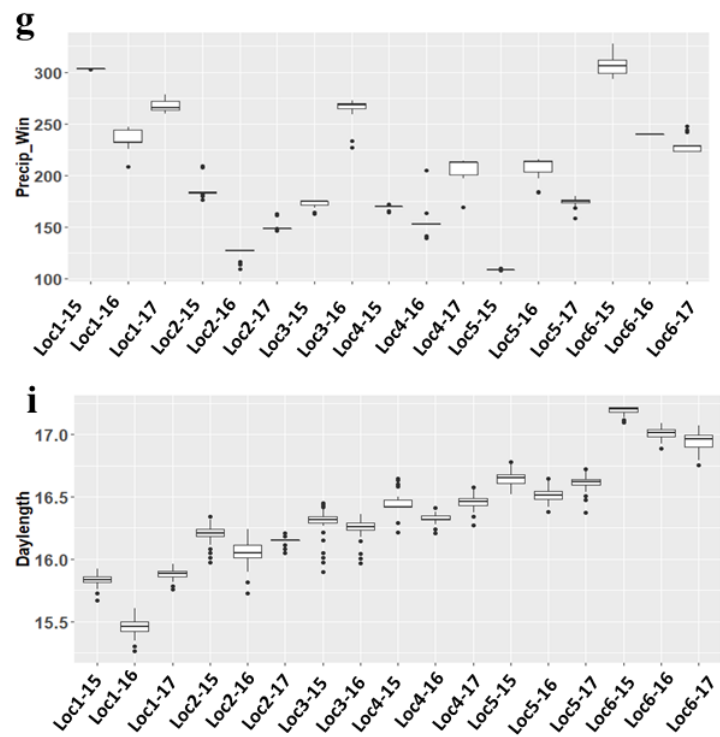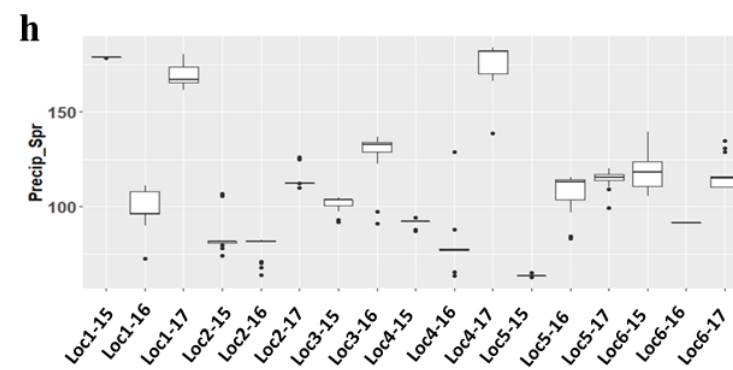

Figure S1

Supplement: Supplementary file 1 — Figure S1 Boxplots showing the measurements of climatic factors per environment according to winter and spring reference dates. Each boxplot in each measurements is based on the scorings per genotype. The mean was considered for the comparison between environments. a, b) The maximal temperature in °C. c, d) The minimal temperature in °C. e, f)The accumulative global radiation in Mj/m2/day. g, h) The accumulative precipitations in mm. i) The daylength in hours(PDF 290 KB) [file 122_2022_4152_MOESM1_ESM.pdf]

2015

2016

2017

Loc1

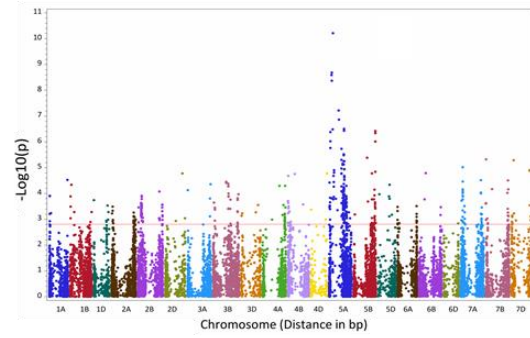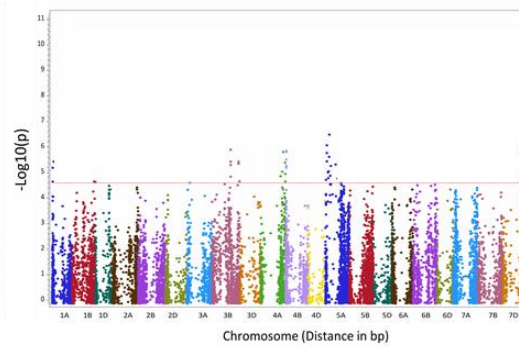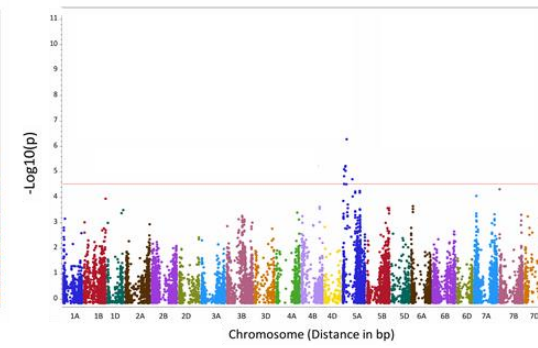

Loc2

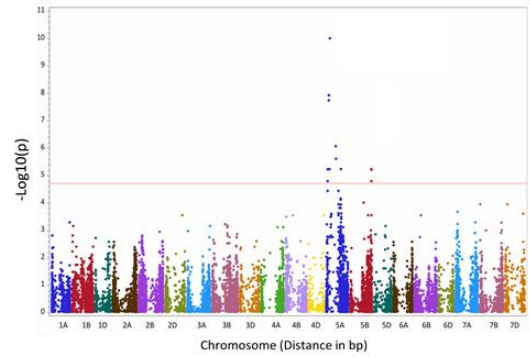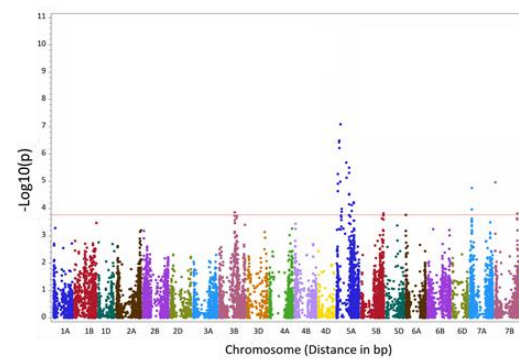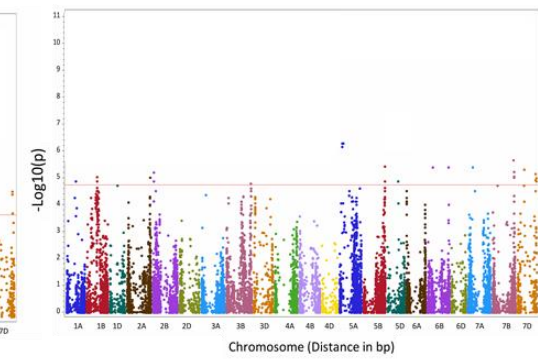

Loc3

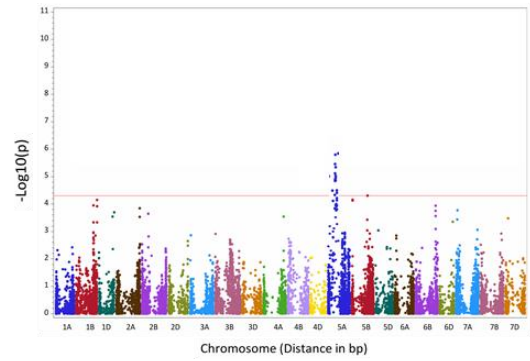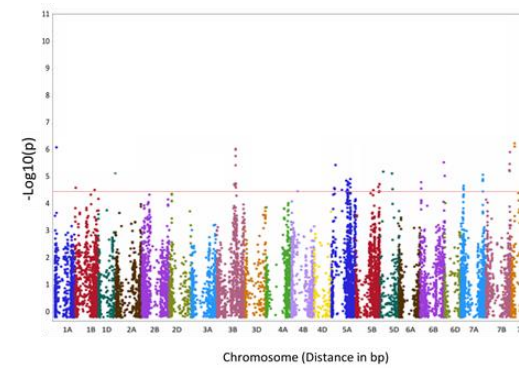

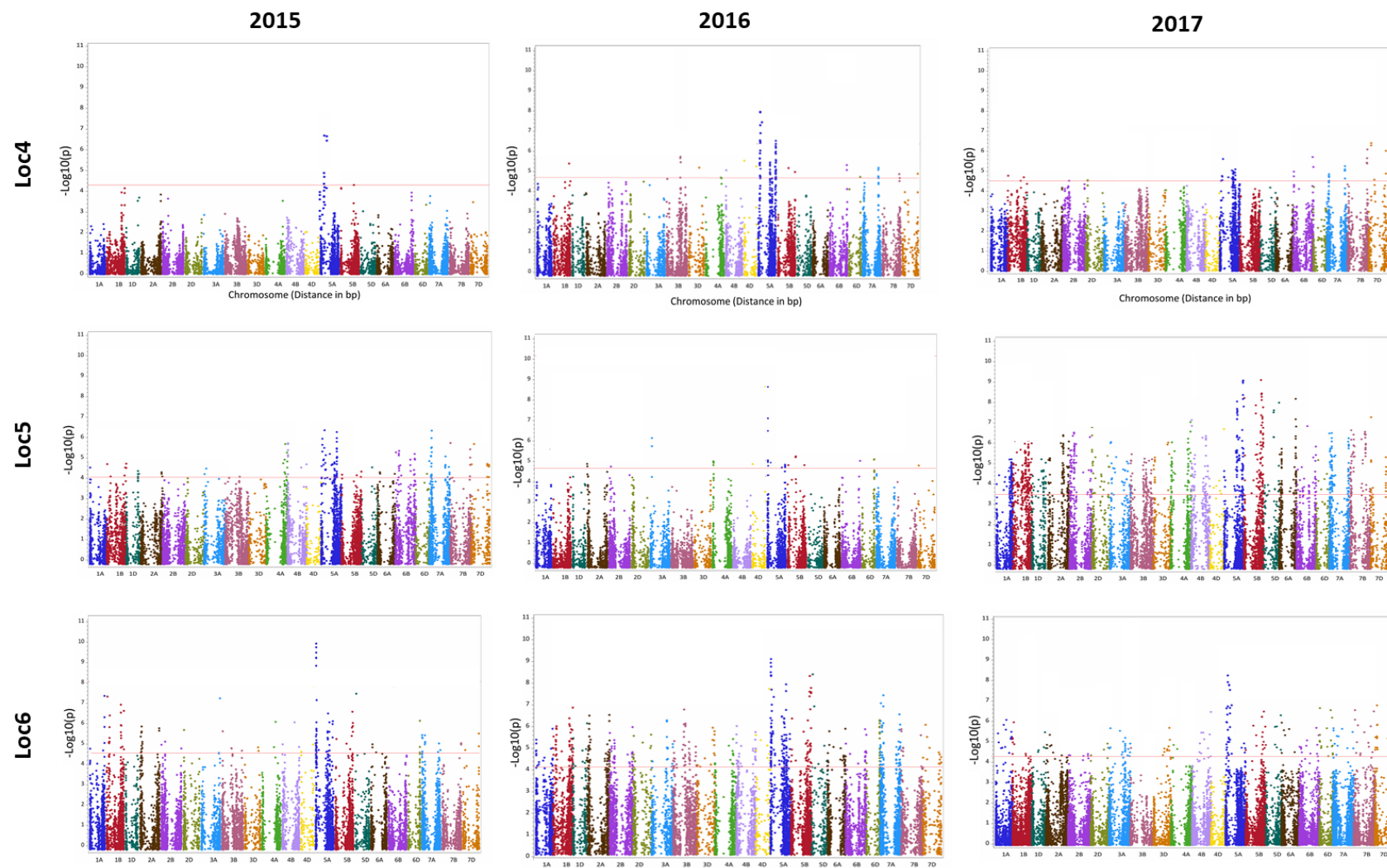

Figure S3

Supplement: Supplementary file 4 — Figure S3 Manhattan plots showing the identified QTL for heading date per environment in panel1 (PDF 1089 KB) [file 122_2022_4152_MOESM4_ESM.pdf]

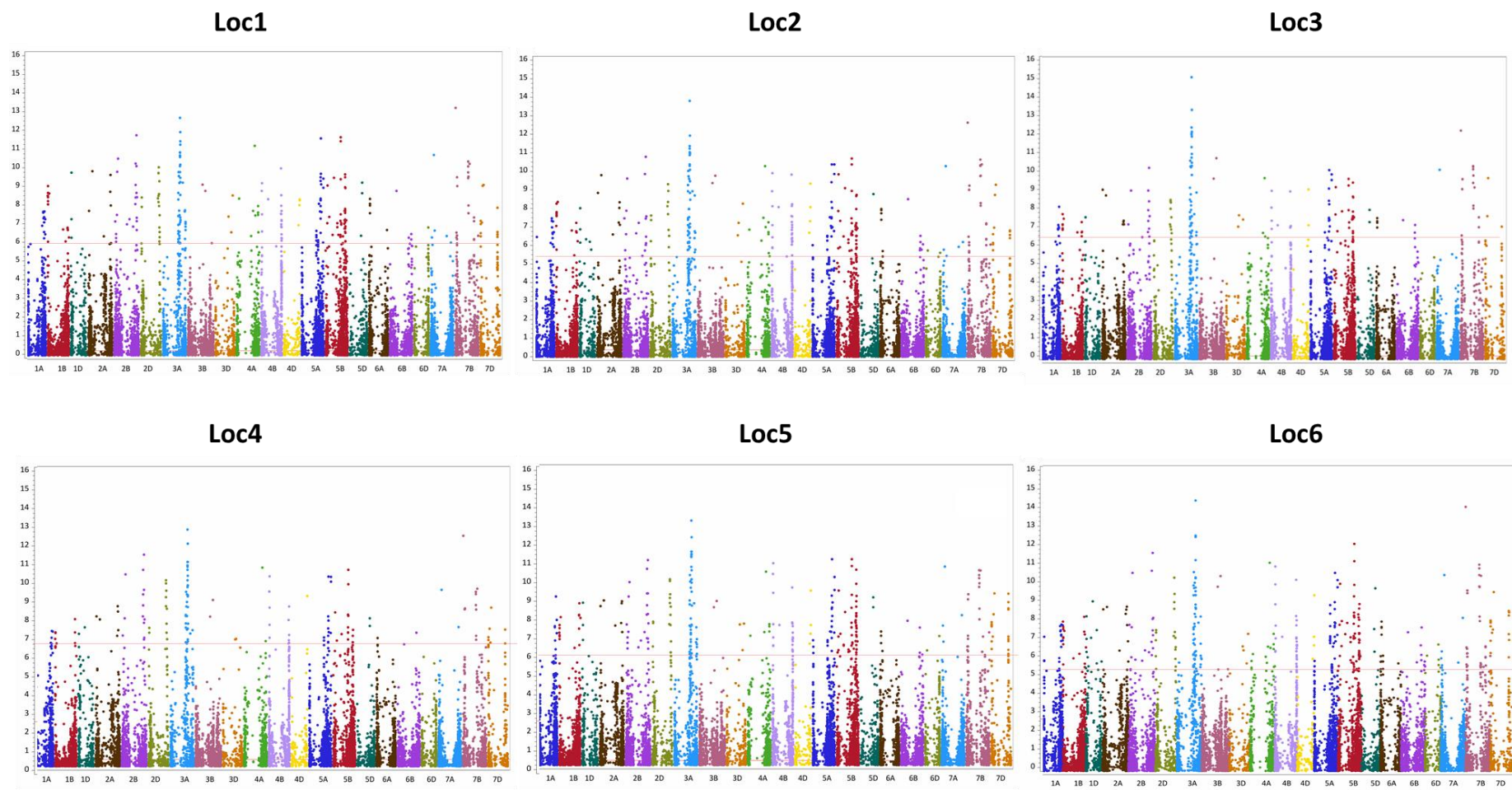

Figure S4

Supplement: Supplementary file 5 — Figure S4 Manhattan plots showing the identified QTL for heading date per environment in panel2. (PDF 351 KB) [file 122_2022_4152_MOESM5_ESM.pdf]

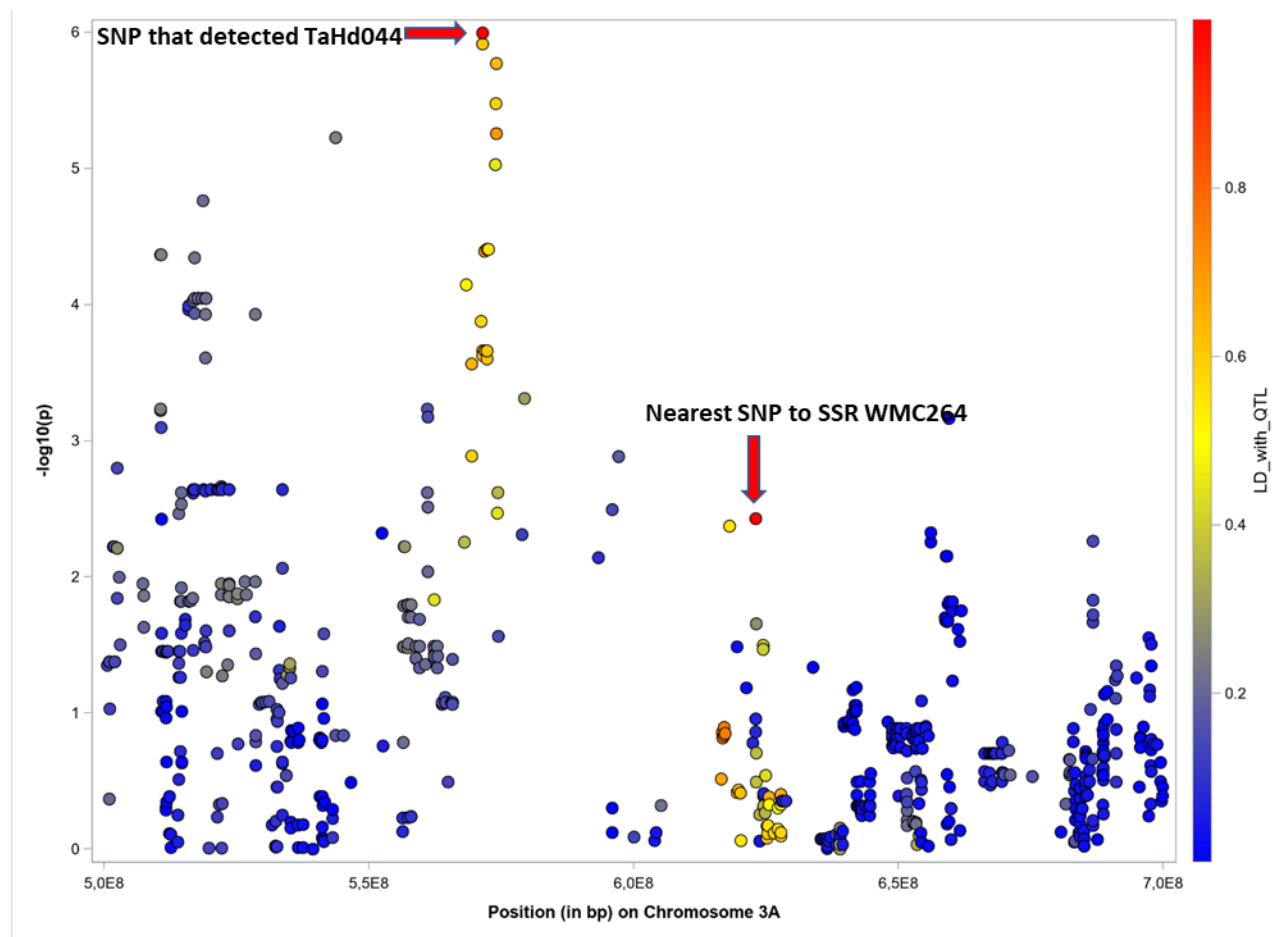

Figure S5

Supplement: Supplementary file 6 — Figure S5 Manhattan plot of genome-wide association study (GWAS) zooming the chromosome 3A interval harboring the TaHd044 QTL. The red dots and arrows indicate the SNP AX-111134276 that detected TaHd044 and the nearest SNP AX-158533114 to SSR WMC264 (Zanke et al.2014). The color scale on the left shows the strength of LD, from blue the weakest to red the strongest. X-axis: the position of the markers in base pairs and y-axis: the LOD values as –log10(p) (PDF 156 KB) [file 122_2022_4152_MOESM6_ESM.pdf]

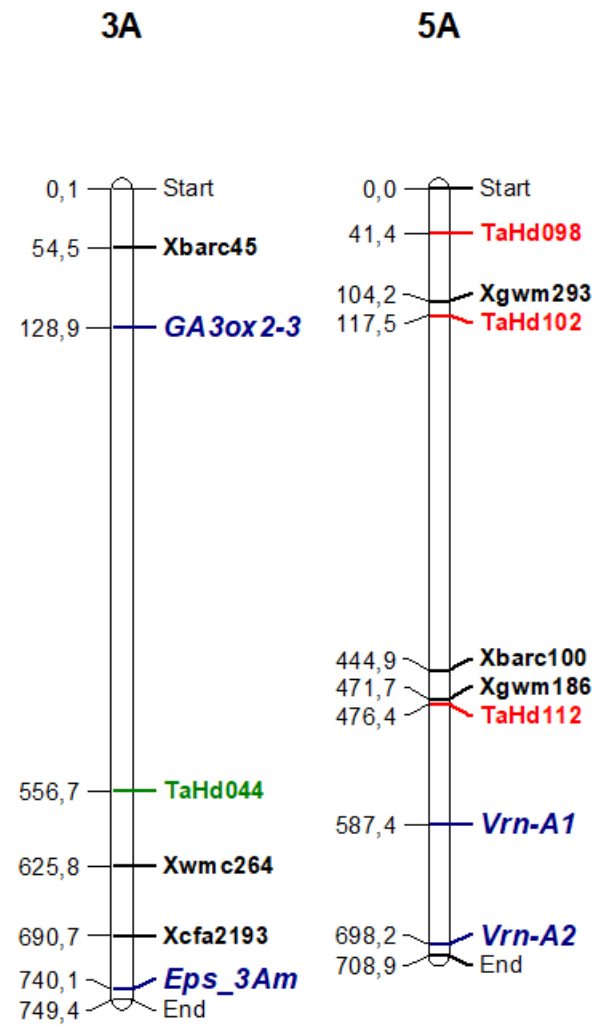

Figure S6

Supplement: Supplementary file 7 — Figure S6 Physical mapping of strongest detected QTL for heading date trait using panel1 (marker in red color) and panel2 (marked in green color) on chromosomes 5A and 3A, respectively (PDF 40 KB) [file 122_2022_4152_MOESM7_ESM.pdf]
