# Supplementary material for: Identification of QTLs for wheat heading time across multiple-environments
Source: Theor Appl Genet. 2022 Jul 1;135(8):2833–48. doi: 10.1007/s00122-022-04152-6 (PMC9325850; doi:10.1007/s00122-022-04152-6)
Supplement: Supplementary file 2 — FigureS2a Correlation matrix showing Peasron correlation coefficients between 17 environments using heading scores based on winter (HD_Win) and spring (HD_Spr) reference dates (PDF 68 KB) [file 122_2022_4152_MOESM2_ESM.pdf]

HD_Win	Loc1_2015	Loc1_2016	Loc1_2017	Loc2_2015	Loc2_2016	Loc2_2017	Loc3_2015	Loc3_2016	Loc4_2015	Loc4_2016	Loc4_2017	Loc5_2015	Loc5_2016	Loc5_2017	Loc6_2015	Loc6_2016	Loc6_2017
Loc1_2015	1																
Loc1_2016	0.71***	1															
Loc1_2017	0.78***	0.70***	1														
Loc2_2015	0.71***	0.60***	0.73***	1													
Loc2_2016	0.77***	0.69***	0.80***	0.75***	1												
Loc2_2017	0.79***	0.69***	0.82***	0.93***	0.94***	1											
Loc3_2015	0.61***	0.49***	0.59***	0.57***	0.60***	0.63***	1										
Loc3_2016	0.65***	0.64***	0.63***	0.67***	0.67***	0.71***	0.59***	1									
Loc4_2015	0.45***	0.43***	0.51***	0.58***	0.56***	0.61***	0.38***	0.42***	1								
Loc4_2016	0.53***	0.46***	0.58***	0.58***	0.70***	0.69***	0.54***	0.58***	0.58***	1							
Loc4_2017	0.65***	0.55***	0.72***	0.65***	0.74***	0.75***	0.57***	0.60***	0.53***	0.62***	1						
Loc5_2015	0.67***	0.56***	0.74***	0.69***	0.75***	0.77***	0.58***	0.59***	0.61***	0.62***	0.64***	1					
Loc5_2016	0.62***	0.60***	0.65***	0.56***	0.66***	0.65***	0.41***	0.55***	0.37***	0.33***	0.50***	0.60***	1				
Loc5_2017	0.74***	0.64***	0.81***	0.67***	0.78***	0.78***	0.65***	0.65***	0.48***	0.57***	0.70***	0.65***	0.57***	1			
Loc6_2015	0.63***	0.55***	0.62***	0.63***	0.72***	0.73***	0.61***	0.61***	0.48***	0.67***	0.59***	0.63***	0.48***	0.60***	1		
Loc6_2016	0.53***	0.46***	0.61***	0.58***	0.56***	0.61***	0.55***	0.62***	0.41***	0.44***	0.52***	0.49***	0.50***	0.65***	0.45***	1	
Loc6_2017	0.68***	0.55***	0.71***	0.71***	0.79***	0.80***	0.55***	0.59***	0.51***	0.64***	0.64***	0.68***	0.52***	0.71***	0.72***	0.47***	1

HD_Spr	Loc1_2015	Loc1_2016	Loc1_2017	Loc2_2015	Loc2_2016	Loc2_2017	Loc3_2015	Loc3_2016	Loc4_2015	Loc4_2016	Loc4_2017	Loc5_2015	Loc5_2016	Loc5_2017	Loc6_2015	Loc6_2016	Loc6_2017
Loc1_2015	1																
Loc1_2016	0.70***	1															
Loc1_2017	0.78***	0.70***	1														
Loc2_2015	0.71***	0.60***	0.73***	1													
Loc2_2016	0.77***	0.68***	0.80***	0.75***	1												
Loc2_2017	0.76***	0.71***	0.86***	0.79***	0.81***	1											
Loc3_2015	0.62***	0.49***	0.59***	0.57***	0.60***	0.59***	1										
Loc3_2016	0.64***	0.64***	0.63***	0.66***	0.66***	0.70***	0.60***	1									
Loc4_2015	0.46***	0.43***	0.51***	0.59***	0.57***	0.59***	0.38***	0.43***	1								
Loc4_2016	0.53***	0.46***	0.58***	0.58***	0.70***	0.60***	0.55***	0.58***	0.58***	1							
Loc4_2017	0.65***	0.55***	0.72***	0.65***	0.75***	0.70***	0.57***	0.61***	0.53***	0.62***	1						
Loc5_2015	0.66***	0.56***	0.74***	0.69***	0.75***	0.74***	0.58***	0.58***	0.62***	0.61***	0.64***	1					
Loc5_2016	0.61***	0.61***	0.65***	0.55***	0.65***	0.64***	0.42***	0.54***	0.38***	0.31***	0.50***	0.60***	1				
Loc5_2017	0.74***	0.64***	0.81***	0.67***	0.78***	0.78***	0.65***	0.65***	0.49***	0.57***	0.70***	0.65***	0.58***	1			
Loc6_2015	0.62***	0.54***	0.62***	0.63***	0.72***	0.61***	0.61***	0.60***	0.48***	0.66***	0.60***	0.63***	0.47***	0.60***	1		
Loc6_2016	0.53***	0.46***	0.60***	0.58***	0.56***	0.58***	0.56***	0.62***	0.42***	0.44***	0.52***	0.48***	0.50***	0.64***	0.45***	1	
Loc6_2017	0.67***	0.55***	0.71***	0.70***	0.78***	0.70***	0.55***	0.58***	0.52***	0.63***	0.64***	0.68***	0.51***	0.71***	0.71***	0.46***	1

Figure S2
