# Supplementary material for: Identification of QTLs for wheat heading time across multiple-environments
Source: Theor Appl Genet. 2022 Jul 1;135(8):2833–48. doi: 10.1007/s00122-022-04152-6 (PMC9325850; doi:10.1007/s00122-022-04152-6)
Supplement: Supplementary file 3 — Figure S2b Geographical heatmap summarizing the correlation between the climatic factors and HD (PDF 68 KB) [file 122_2022_4152_MOESM3_ESM.pdf]

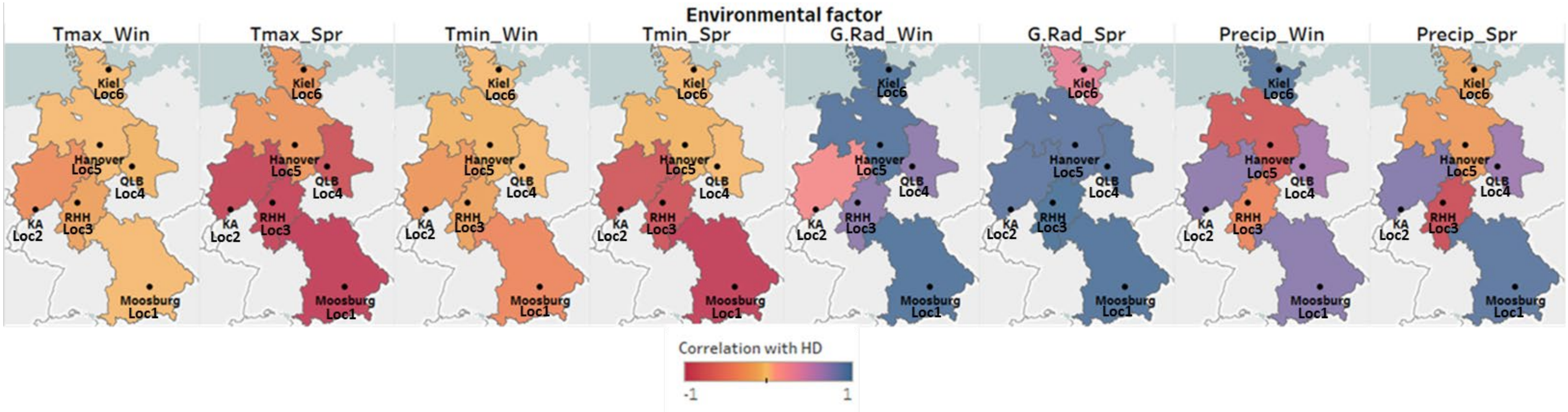

Location	Tmax_Win	Tmax_Spr	Tmin_win	Tmin_Spr	G.Rad_Win	G.Rad_Spr	Precip_Win	Precip_Spr
Loc1	-0.10*	-0.98**	-0.35**	-0.98**	0.99**	0.99**	0.77**	0.95**
Loc2	-0.31**	-0.91**	-0.18**	-0.79**	0.2*	0.92**	0.74**	0.78**
Loc3	-0.16**	-0.93**	-0.23**	-0.81**	0.74**	1**	-0.35**	-0.88**
Loc4	-0.04**	-0.83**	0	0	0.75**	0.94**	0.64**	0.68**
Loc5	0.02**	-0.25**	-0.04	-0.04*	0.97**	0.92**	-0.75**	-0.21**
Loc6	0.06*	-0.26**	0	0	0.99**	0.33**	0.97**	-0.13**

Figure S2b: Geographical heatmap summarizing the correlation between the climatic factors (The minimal and maximal temperature, the global radiation and the amount of precipitations) and HD based on winter and spring records per location. Each correlation was run separately including HD scores of three years per location. (*), (**) indicate level of significance $p < 0.1$ and $p < 0.01$, respectively. Loc1: Moosburg, Loc2: Klein-Altendorf (KA), Loc3: RHH, Loc4; Quedlinburg, Loc5: Hannover, Loc6: Kiel
